# A Phase I Trial of DFMO Targeting Polyamine Addiction in Patients with Relapsed/Refractory Neuroblastoma

**DOI:** 10.1371/journal.pone.0127246

**Published:** 2015-05-27

**Authors:** Giselle L. Saulnier Sholler, Eugene W. Gerner, Genevieve Bergendahl, Robert B. MacArthur, Alyssa VanderWerff, Takamaru Ashikaga, Jeffrey P. Bond, William Ferguson, William Roberts, Randal K. Wada, Don Eslin, Jacqueline M. Kraveka, Joel Kaplan, Deanna Mitchell, Nehal S. Parikh, Kathleen Neville, Leonard Sender, Timothy Higgins, Masao Kawakita, Kyoko Hiramatsu, Shun-suke Moriya, André S. Bachmann

**Affiliations:** 1 Helen DeVos Children’s Hospital, Grand Rapids, Michigan, United States of America; 2 College of Human Medicine, Michigan State University, Grand Rapids, Michigan, United States of America; 3 Cancer Prevention Pharmaceuticals, Tucson, Arizona, United States of America; 4 Medical Biostatistics, University of Vermont, Burlington, Vermont, United States of America; 5 Department of Microbiology and Molecular Genetics, University of Vermont College of Medicine, Burlington, Vermont, United States of America; 6 Cardinal Glennon Children's Hospital, St. Louis, Missouri, United States of America; 7 University of California San Diego School of Medicine and Rady Children's Hospital, San Diego, California, United States of America; 8 Kapiolani Medical Center for Women and Children, Honolulu, Hawaii, United States of America; 9 Arnold Palmer Hospital for Children, Orlando, Florida, United States of America; 10 Medical University of South Carolina, Charleston, South Carolina, United States of America; 11 Levine Children's Hospital, Charlotte, North Carolina, United States of America; 12 Connecticut Children's Medical Center, Hartford, Connecticut, United States of America; 13 Children's Mercy Hospitals and Clinics, Kansas City, Missouri, United States of America; 14 Children’s Hospital of Orange County, Orange, California, United States of America; 15 Tokyo Metropolitan Institute of Medical Science, Tokyo, Japan; 16 University of Hawaii at Hilo, The Daniel K. Inouye College of Pharmacy, Hilo, Hawaii, United States of America; National Cancer Institute, UNITED STATES

## Abstract

**Background:**

Neuroblastoma (NB) is the most common cancer in infancy and most frequent cause of death from extracranial solid tumors in children. Ornithine decarboxylase (ODC) expression is an independent indicator of poor prognosis in NB patients. This study investigated safety, response, pharmacokinetics, genetic and metabolic factors associated with ODC in a clinical trial of the ODC inhibitor difluoromethylornithine (DFMO) ± etoposide for patients with relapsed or refractory NB.

**Methods and Findings:**

Twenty-one patients participated in a phase I study of daily oral DFMO alone for three weeks, followed by additional three-week cycles of DFMO plus daily oral etoposide. No dose limiting toxicities (DLTs) were identified in patients taking doses of DFMO between 500-1500 mg/m2 orally twice a day. DFMO pharmacokinetics, single nucleotide polymorphisms (SNPs) in the ODC gene and urinary levels of substrates for the tissue polyamine exporter were measured. Urinary polyamine levels varied among patients at baseline. Patients with the minor T-allele at rs2302616 of the ODC gene had higher baseline levels (p=0.02) of, and larger decreases in, total urinary polyamines during the first cycle of DFMO therapy (p=0.003) and had median progression free survival (PFS) that was over three times longer, compared to patients with the major G allele at this locus although this last result was not statistically significant (p=0.07). Six of 18 evaluable patients were progression free during the trial period with three patients continuing progression free at 663, 1559 and 1573 days after initiating treatment. Median progression-free survival was less among patients having increased urinary polyamines, especially diacetylspermine, although this result was not statistically significant (p=0.056).

**Conclusions:**

DFMO doses of 500-1500mg/m2/day are safe and well tolerated in children with relapsed NB. Children with the minor T allele at rs2302616 of the ODC gene with relapsed or refractory NB had higher levels of urinary polyamine markers and responded better to therapy containing DFMO, compared to those with the major G allele at this locus. These findings suggest that this patient subset may display dependence on polyamines and be uniquely susceptible to therapies targeting this pathway.

**Trial Registration:**

Clinicaltrials.gov NCT#01059071

## Introduction

Neuroblastoma (NB) is a deadly childhood cancer that arises from neural crest cells of the sympathetic nervous system. The average age at diagnosis is 17 months and 50–60% of patients present with metastatic disease. NB is a heterogeneous disease, with varied risk groups [1]. Up to 45% of patients are in a high-risk category that includes patients with MYCN amplification or other adverse clinicopathologic features. Despite advances in treatments that include chemotherapy, surgery, radiation, high dose chemotherapy with stem cell rescue, antibody and biologic-based therapy, the overall long-term survival of patients with high risk disease remains poor at approximately 50%. Approximately 20% of patients in this high-risk group fail to respond adequately to chemotherapy and develop progressive or refractory disease. Those which complete upfront therapy will have a >35% risk of relapse [2–4]. As such new therapies for patients with relapsed or refractory NB are sorely needed.

In 2004, we began investigating Difluoromethylornithine (DFMO) for the treatment of high-risk NB [5]. DFMO is an enzyme-activated inhibitor of ornithine decarboxylase (ODC) and ODC is a rate-limiting enzyme of polyamine biosynthesis. Our preclinical studies with DFMO showed that polyamine depletion is an effective therapeutic strategy in NB [6]. We found that DFMO alters the polyamine-regulated p27Kip1/Rb signaling pathway that leads to G1 cell cycle arrest and prevents NB migration/invasion of cells [6–8]. We and other groups independently validated the epidemiological and laboratory evidence that indicated that ornithine decarboxylase (ODC) and several other genes in the polyamine pathway were transcriptional targets of MYCN [9–11]. Our observations with DFMO were confirmed in vivo by two groups using the TH-MYCN transgenic NB mouse model [9, 10]. We further demonstrated that ODC expression is a negative risk factor for NB independent of MYCN amplification [11]. ODC gene expression is directly activated by MYCN, and in a subset of patients is co-amplified with MYCN [9], suggests that MYCN gene amplification leads to high ODC expression and subsequent high polyamine levels which contribute to the malignant phenotype and the maintenance of NB tumorigenesis [12–18].

Single nucleotide polymorphisms (SNPs) in the ODC gene have been associated with risk of specific cancers [19–21]. The minor A allele at rs2302615 in the ODC gene was found to be a risk allele for survival in patients with prior colorectal cancer [22], but a protective allele in patients with NB [23]. The SNP at rs2302615 affects binding to the surrounding DNA elements of e-box transcription factors [19, 22, 23], which have been found to interact with transcription factors acting at an upstream SNP (rs2302616) [24]. The minor T allele at rs2302616 disrupts a G-quadraplex structure in the ODC gene, increases ODC promoter activity and is associated with increased putrescine content in rectal tissues from patients with risk of colorectal cancer [24, 25]. Patients in a colorectal adenoma prevention trial with this genotype also display maximal response to a combination of agents targeting the polyamine pathway [25], suggesting that the minor T-allele at rs2302616 may convey a “polyamine addiction” phenotype.

While the importance of ODC and polyamines in tumor growth has been well established [26, 27], the usefulness of DFMO in the treatment of pediatric NB had not been considered until recently [5, 6] and this is the first trial to evaluate DFMO clinically in NB patients. Orally administered DFMO is an experimental therapy that has never received regulatory approval for any indication. High-dose Intravenous (IV) DFMO received regulatory approvals in 1990 for first-line treatment of West African sleeping sickness (trypanosomiasis), and is used by the World Health Organization in combination with nifurtimox, also referred to as Nifurtimox-Eflornithine-Combination-Therapy (NECT) [28, 29]. Topical DFMO is the active component of a commercial therapy for hirsutism (excess facial hair) [30].

The primary aim of this phase I clinical trial was to study the safety of the ODC inhibitor difluoromethylornithine (DFMO) alone and in combination with a cytotoxic chemotherapeutic drug in pediatric patients with refractory or recurrent NB. Etoposide was chosen for the combination, as it has reported efficacy in this patient group [31] and is synergistic with DFMO in some cell models [32]. The secondary aims were to investigate the activity, pharmacokinetics and genetic and metabolic factors associated with ODC.

## Patients and Methods

The protocol for this trial and supporting TREND checklist are available as supporting information; see S1 TREND Checklist and S1 Protocol.

### Patient Eligibility

The supporting TREND checklist for this trial is available as supporting information; see S1 TREND Checklist. Patients were enrolled onto the Neuroblastoma and Medulloblastoma Translational Research Consortium (NMTRC) 002 study from March 2010 to October 2012. To be eligible for this study, subjects had to fulfill the following criteria: (a) age 0–21 years at the time of diagnosis; (b) histologic verification at either the time of original diagnosis or relapse of NB; (c) disease status verified as refractory or relapsed NB; (d) measurable disease based on measurable tumor (>10 mm by CT or MRI), positive MIBG and abnormal urinary catecholamine levels or positive bone marrow biopsy/aspirate; (e) disease state was one for which there was no known curative therapy; (f) negative urine pregnancy test for female subjects of child bearing potential (onset of menses or ≥ 13 years of age); (g) adequate liver function as defined by AST and ALT <10x normal. Exclusion criteria were life expectancy <2 months, Lansky score <30%, or subjects who were concurrently receiving another investigational drug or anticancer agent. Subjects had to be fully recovered from the effects of prior chemotherapy (hematological and bone marrow suppression effects). Subjects were excluded if they had an uncontrolled infection until the infection was controlled. Subjects who were not able to comply with the safety monitoring requirements of the study were also excluded. This trial was approved by the Western Institutional Review Board as well as by local Institutional Review Boards at each enrolling site as follows; University of Vermont Committees on Human Research, The Spectrum Health Institutional Review Board, M. D. Anderson-Orlando Institutional Review Board, The Institutional Review Board of Carolinas HealthCare System, and Children’s Hospital of Orange County Institutional Review Board. Written informed consent was obtained from the patients' parent(s) or guardian(s), and patients provided written assent when appropriate, prior to study entry. ClinicalTrials.gov Identifier: NCT01059071.

### Patient Characteristics

Twenty-one subjects with refractory or recurrent NB were enrolled in this study between March 2010 and October 2012. The subject characteristics are shown in Table 1. Every subject had previously received standard therapy for their disease and had relapsed or was refractory to therapy. The median age was 9 years old, with a range of 1–17 years old. Additional enrollment characteristics including number and type of previous relapse treatments, MYCN status, and disease status at study entry can be found in S1 Table.

**Table 1:** Characteristics of patients enrolled in NMTRC 002.

**Enrollment**	N
Total Enrolled	21
Total Received Drug	21
**Evaluable**	N (%)
Efficacy Evaluable	18 (86)
Safety Evaluable	21 (100)
**Age**	Years
Mean	8.75
Median	9
**Sex**	N (%)
Male	14 (67)
Female	7 (33)
**Race**	N (%)
Caucasian	14 (67)
Hispanic	3 (14)
Black or African American	2 (9.5)
More than one race or unknown	2 (9.5)

### Study Design and Treatment

**Fig 1:**
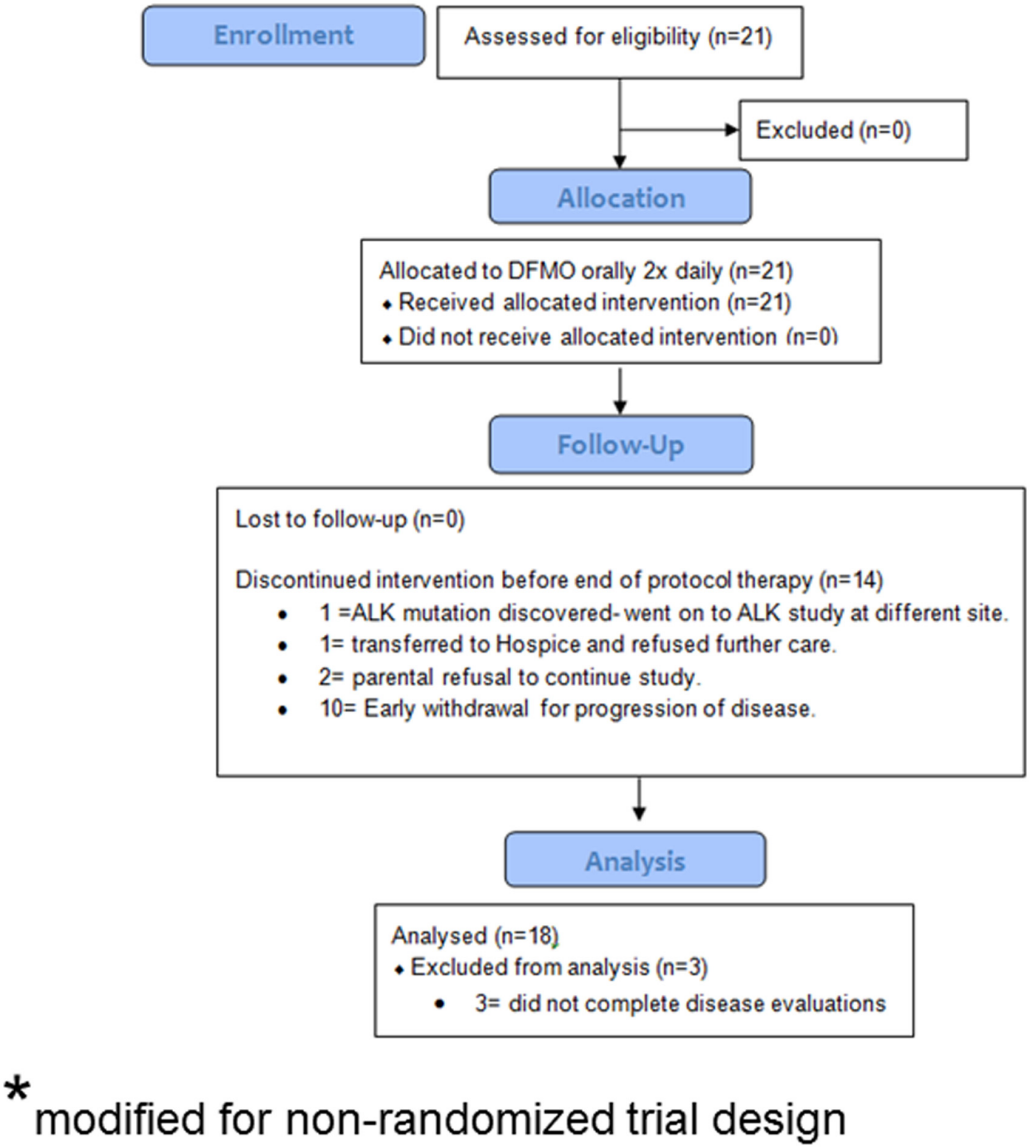
NMTRC002 CONSORT Flow Diagram- modified for non-randomized trial design.

**Fig 2:**
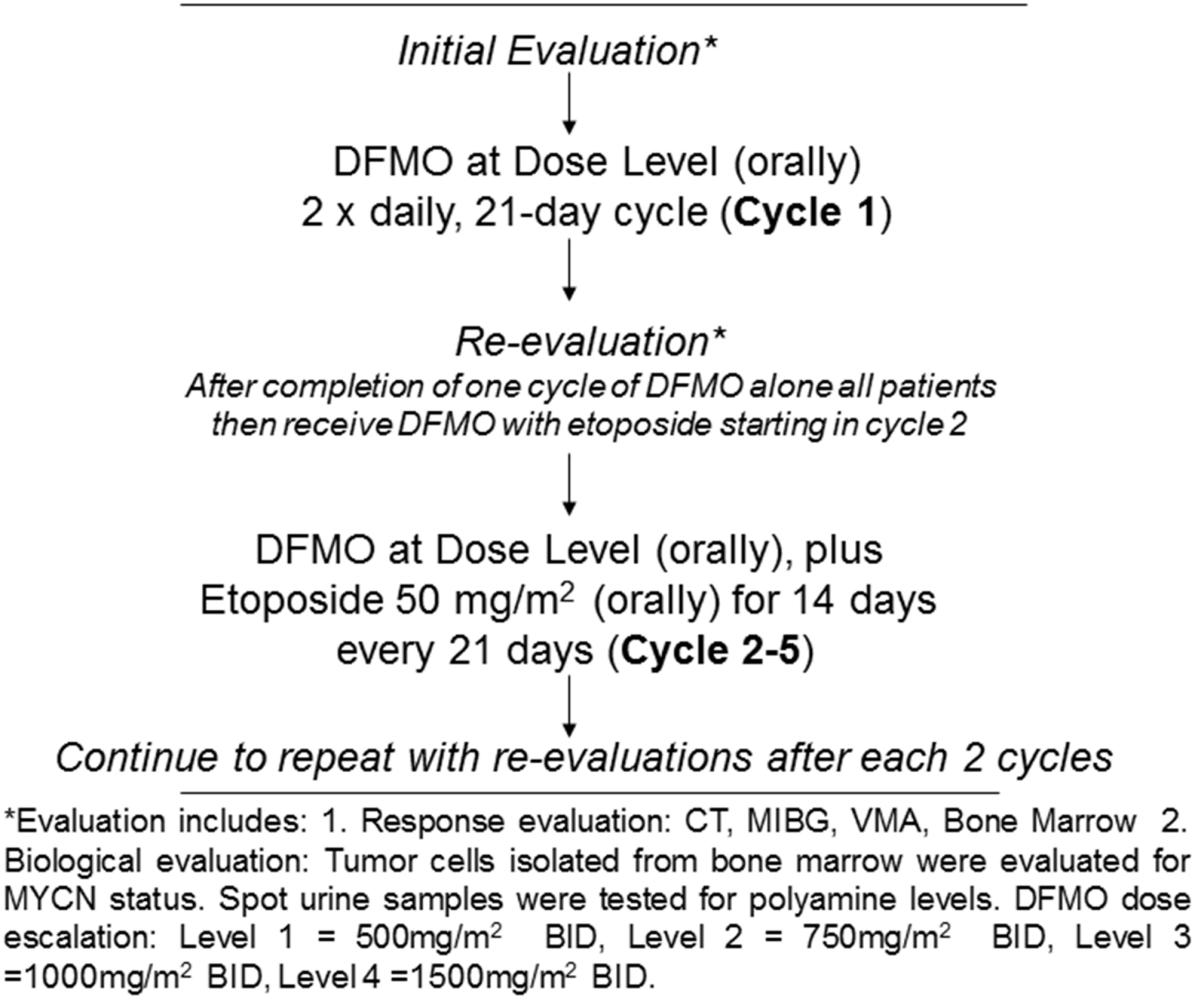
Flowchart of NMTRC 002—Safety Study for Refractory or Relapsed Neuroblastoma With DFMO Alone and in Combination With Etoposide.

The NMTRC 002 CONSORT flow diagram which has been modified for a non-randomized trial is shown (Fig 1) along with the study design flowchart (Fig 2). This trial was a standard 3+3 Phase I dose escalation design. In order to address the safety issue, patient replacement was allowed if a patient withdrew from the trial for non-drug related reasons prior to completion of 2 cycles of the protocol. Patients displaying a clinical response were allowed to remain on treatment until disease progression occurred or mutual decision of their physician and parents. Subjects were enrolled at one of four escalating doses.

Dose limiting toxicities (DLT) for single agent DFMO were evaluated in Cycles 1 (DFMO alone) and Cycle 2 (DFMO and etoposide combination) for determination of the maximum tolerated dose (MTD) of this treatment. DLT was defined as patients experiencing any toxicity specified as Grade 4 neutropenia or thrombocytopenia that persists for 7 days or longer off study drug, Grade 3 elevation of transaminases that persists for 7 days or longer off study drug, or any other Grade 3 non-hematologic toxicity, excluding alopecia or inadequately treated nausea, vomiting, diarrhea. The MTD was defined as the dose level below which DLTs are seen in ≥ two of six subjects dosed.

Given the tolerability of DFMO in adult studies it is possible that these endpoints, DLT and MTD, may not be met at these doses chosen. These doses will be evaluated for biologic activity through measurement of urinary polyamines to determine if higher dosing is required in future studies.

### Drug Formulation and Administration

Subjects received single agent DFMO administered orally on Days 1–21 of the first 21-day cycle. DFMO was supplied as a powder that was dissolved in juice or water prior to administration. The starting dose was 500 mg/m^2^ PO BID (Dose Level 1). Dose escalation took place in a standard 3+3 design, in which doses increased by approximately 20 to 25% in successive 3-subject cohorts. These additional doses included Dose Level 2 at 750 mg/m2 PO BID, Dose Level 3 at 1000 mg/m2 PO BID, and Dose Level 4 at 1500 mg/m2 PO BID. Enrollment of the next cohort occurred after the entire previous cohort had completed both cycles 1 (single agent) and 2 (combination) of treatment without any dose limiting toxicity (DLT), as reviewed by the Data and Safety Monitoring Committee. After the first cycle of single agent DFMO all patients received DFMO in combination with etoposide in cycles 2–5. During these cycles, subjects continued to receive DFMO at the dose received during cycle 1 in addition to oral Etoposide at 50 mg/m^2^/dose (rounded to the nearest 50 mg) once daily for the first 14 days of Cycles 2–5. The final cohort of DFMO received an additional 3 enrollments as a confirmation cohort, so that six subjects received a maximum of 1500 mg/m^2^ BID dose of DFMO.

The etoposide was discontinued in some patients after cycle 5 due to the concern for risk of secondary leukemia. The decision was made by the site physician/primary investigator and patient family.

### Patient safety and treatment response evaluation

Weekly monitoring for treatment related toxicities included a physical exam, vital signs (temperature, pulse rate, and sitting blood pressure) CBC, AST/ALT, LDH, bilirubin, electrolytes, BUN, creatinine, review and recording of concomitant medications, and monitoring of AE’s with a review of concurrent illnesses. In addition, Lansky or ECOG score and urine catecholamines were measured prior to every 21 day cycle. An audiogram was performed at the end of cycles 1, 3, and 5. Subjects without bone marrow metastases were required to have adequate bone marrow function as defined by ANC > 500/μl and platelets > 50,000/μl before starting chemotherapy. Clinical and laboratory adverse events were graded according to the NCI-common terminology criteria for adverse events (CTCAE) version 3.0.

Tumor and clinical responses were monitored as secondary endpoints. Eighteen subjects were evaluated for efficacy. This study used the (RECIST) Response Evaluation Criteria measurements in Solid Tumor from the NCI modified for pediatrics as well as MIBG or PET and bone marrow response. Tumor assessments/imaging studies were obtained at baseline >7 days from prior therapy and < 21 days from the start of study therapy. These were repeated at the end of the first cycle and again after every other cycle.

### Pharmacokinetic (PK) Analytical Method and Sample Collection

Patients were consented for all pharmacokinetic sampling and analysis. DFMO analytical methods were performed in compliance with Good Laboratory Practice (GLP), under contract with inVentiv Health Clinique (Quebec, Canada). Briefly, the analyte DFMO and its internal standard were extracted from a 0.025 mL aliquot of human serum. The extracted samples were injected into a liquid chromatograph equipped with an Atlantis Hilic Silica, 50 x 4.6 mm, 3 μm column. The mobile phase A was a mixture of Milli-Q type water with acetonitrile and ammonium acetate. The validated calibration range for this assay was from 50 to 100000 ng/mL. Blood was drawn from patients immediately prior to taking a morning oral DFMO dose during cycles 1 (DFMO alone) and 2 (DFMO + etoposide) and at 0.5, 1, 3 and 6 hours after drug administration. DFMO levels were then assessed in serum obtained from these blood samples. Blood was not collected beyond 6 hours post dose as this trial was conducted on an outpatient basis, and this was judged to be an undue burden on patients.

### ODC Genotype

Patients were consented for genetic analysis in NMTRC 002. ODC rs2302615 and rs2302616 genotypes were determined from blood samples by pyrosequencing methods, under contract with EpigenDx (epigendx.com).

### Urinary Polyamine Levels

Patients were consented for urine analysis in NMTRC 002. Spot urine (first void of the day) was collected on days 1, 8, and 15 of cycle 1 (DFMO only) and frozen at -80°C until analysis of polyamines levels. Polyamines with at least one free primary amine were quantified using reverse-phase high-performance liquid chromatography (HPLC) as previously described [33]. Urinary N^1^,N^12^-diacetylspermine (N^1^,N^12^-Ac_2_Spm or DAS) was determined using the auto DAS reagent kit (Alfresa Pharma Co., Osaka, Japan), according to the manufacturer's instructions. The assay involves the specific binding between a bovine serum albumin-acetylspermine conjugate, as a DAS mimic, and colloidal gold antibody complexes, and has been previously described [34].

### Statistical Methods

Pharmacokinetic parameters C_max_, t_max_ and AUC_0–6_ are presented as the mean and standard deviation of all observed values at each dose level, and were analyzed using SAS (ver. 9.2). Urinary polyamine levels were derived from duplicate measurements of individual samples. Friedman's test for repeated measures analysis of variance was used to assess changes in contents of individual urinary polyamines.

Analyses of associations among PFS, urinary polyamines, and genotype were based on nonparametric methods. These analyses include point estimation, interval estimation, and hypothesis tests. Median progression-free survival was estimated using the Kaplan-Meier method [35]; 95% confidence intervals were estimated based on the cumulative hazard (method *survfit* from the R *survival* [36–38] package). Two-sample progression-free survival difference tests were based on log-rank test [39] (method *survdiff* from the *survival* package). Confidence intervals on the means of polyamine measurements were obtained by bootstrapping [40, 41]. Hypothesis tests on differences of polyamine concentrations were based on the Wilcoxon rank-sum test (method *wilcox*.*test* of the R *stats* [36] package).

## Results

### Patient Enrollment

Three evaluable subjects were enrolled at 500 mg/m2 BID, three evaluable subjects at 750 mg/m2 BID, three evaluable subjects at 1000 mg/m2 BID, and six subjects at 1500 mg/m2 BID. Twenty-one subjects received at least one dose of DFMO as a single agent and were evaluable for safety. Eighteen of those subjects completed cycle 1 and were evaluable for efficacy of DFMO alone. After one cycle of DFMO alone, patients were able to continue the study with DFMO and etoposide combination therapy. Of the initial eighteen patients, fifteen subjects completed at least one additional cycle of DFMO with etoposide and comprise the population evaluable for dose limiting toxicity. Of the eighteen subjects that were evaluable for efficacy, 2 subjects completed 1 cycle, 7 subjects completed 3 cycles, 2 subjects completed 5 cycles, 1 subject completed 7 cycles (cycles 6–7 DFMO alone), 1 subject completed 10 cycles, 1 subject completed 12 cycles (cycles 7–12 DFMO alone), 1 subject completed 15 cycles (cycles 6–15 DFMO alone), 2 subjects completed 17 cycles (on subject cycles 6–17 DFMO alone), and 1 subject completed 43 cycles on study (cycles 7–43 DFMO alone).

### Safety of Oral DFMO and Etoposide

No dose-limiting toxicities (DLTs) or drug related serious adverse events (SAEs) were observed in this study. Study related (possibly, probably and definitely related) toxicities observed during all cycles are summarized in Table 2. Those related to DFMO alone consisted of anemia (n = 3), ANC decrease (n = 2), decreased platelet count (n = 2), ALT increase (n = 1), AST increase (n = 1), anorexia (n = 1), constipation (n = 1), diarrhea (n = 1), infection (conjunctivitis) (n = 1), hypoalbuminemia (n = 1), hypophosphatemia (n = 1), increased GGT (n = 1), sleep disturbance (n = 1), urinary retention (n = 1) and vomiting (n = 1). Six subjects were enrolled in the 1500 mg/m^2^ BID dose and no DLTs were observed. Thus, the dose of DFMO recommended for Phase II evaluation is 1500 mg/m^2^ BID. A maximum tolerated dose (MTD) was not established in this study.

**Table 2:** Study Safety Data: Toxicity of Oral DFMO and Etoposide.

	Maximum grade of toxic effects, Cycle 1				Maximum grade of toxic effects, Cycle 2–43			
	n = 21				n = 17			
	Grade 2	Grade 3	Grade 4	Grade 5	Grade 2	Grade 3	Grade 4	Grade 5
**Hematologic Toxic Effects**								
Anemia	2 (10%)	0	1 (5%)	0	4 (24%)	1 (6%)	0	0
Neutrophil count decrease	1 (5%)	1 (5%)	0	0	3 (18%)	2 (12%)	2 (12%)	0
Platelet count decrease	1 (5%)	1 (5%)	0	0	1 (6%)	1 (6%)	1 (6%)	0
White blood cell decreased	0	0	0	0	0	0	1 (6%)	0
**Non-hematologic Toxic Effects**								
ALT elevation	1 (5%)	0	0	0	1 (6%)	0	0	0
Anorexia	0	1 (5%)	0	0	0	0	0	0
AST elevation	0	1 (5%)	0	0	1 (6%)	1 (6%)	0	0
Conjunctivitis	1 (5%)	0	0	0	0	0	0	0
Constipation	1 (5%)	0	0	0	1 (6%)	0	0	0
Diarrhea	1 (5%)	0	0	0	0	0	0	0
GGT elevation	1 (5%)	0	0	0	0	0	0	0
Hypoalbuminemia	1 (5%)	0	0	0	0	0	0	0
Hypophosphatemia	1 (5%)	0	0	0	0	0	0	0
Infection, sinus	0	0	0	0	1 (6%)	0	0	0
Mouth pain	0	0	0	0	1 (6%)	0	0	0
Nausea	0	0	0	0	1 (6%)	0	0	0
Neuropathy	0	0	0	0	1 (6%)	0	0	0
Pain	0	0	0	0	1 (6%)	0	0	0
Rash	0	0	0	0	1 (6%)	0	0	0
Sleep disturbance	1 (5%)	0	0	0	0	0	0	0
Urinary retention	1 (5%)	0	0	0	0	0	0	0
Vomiting	1 (5%)	0	0	0	0	0	0	0

Percentages are calculated as number of patients with an event divided by number of patients in group that received drug.

ALT = alanine aminotransferase

AST = aspartate aminotransferase

GGT = gamma-glutamyl transpeptidase

Adverse events attributed (possibly, probably, or definitely) to DFMO (cycle 1) or DFMO/etoposide (cycles 2–43) across all dose levels

### Pharmacokinetics of DFMO in children with NB

**Fig 3:**
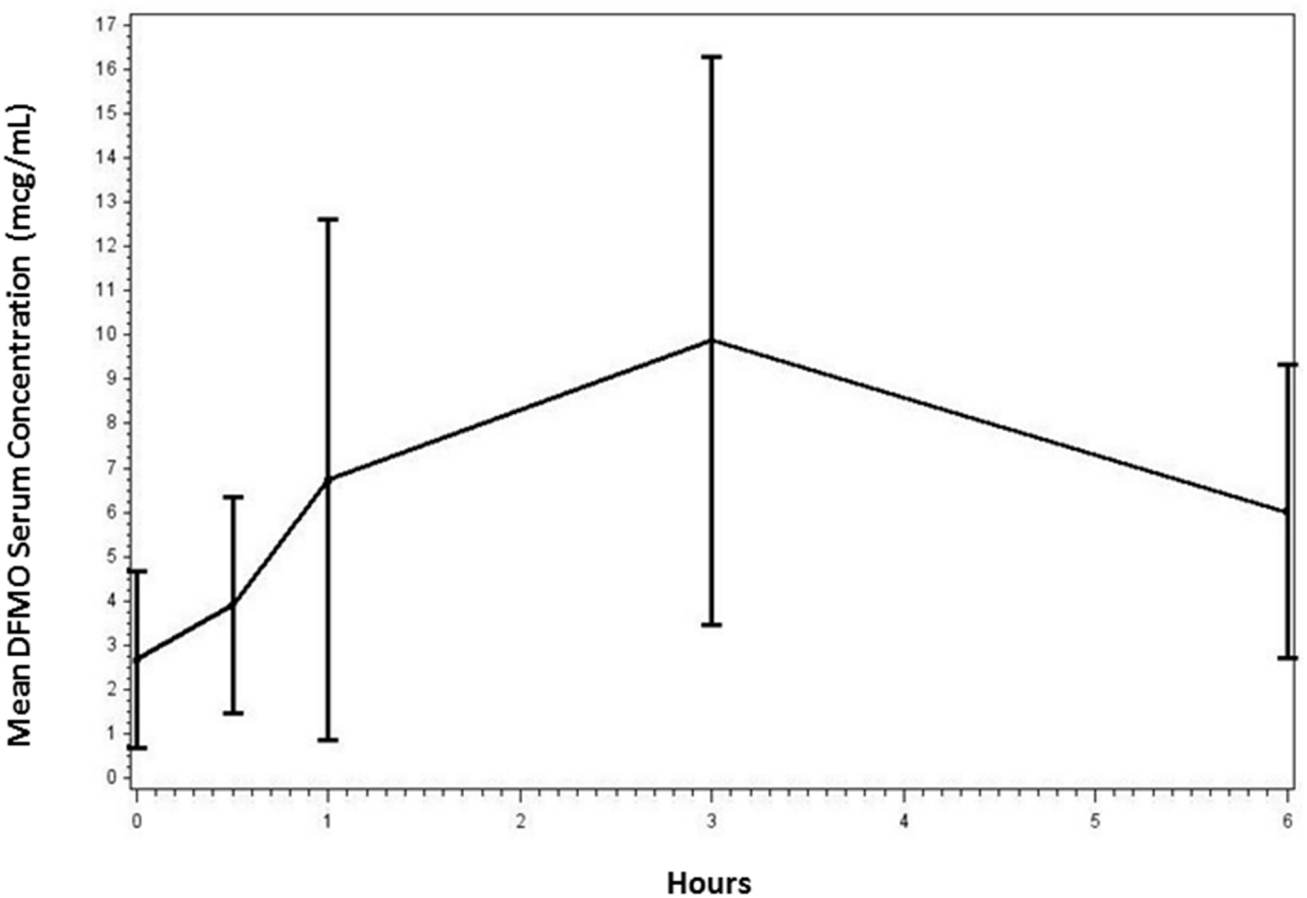
Serum DFMO concentration versus time measurements for three patients receiving 750 mg/m^2^ PO BID during cycle 1 of therapy.

DFMO serum measurements were performed in all 21 patients. Samples were collected from patients prior to, and again at times 0.5, 1, 3 and 6 hours following drug administration on days 1 and 8 of the first cycle. DFMO serum samples were also collected from selected patients in the higher dose groups (750, 1000, 1500 mg/m^2^) during cycle 2. The serum DFMO concentrations (mean and sd) in all patients receiving 750 mg/m^2^ (mean ± standard deviation) is shown in Fig 3. DFMO doses were administered orally twice daily over a 21 day cycle. Subsequent cycles commenced the day following the last day of the previous cycle. Maximum DFMO concentrations, relative to dose, are reported in Table 3. Overall average serum DFMO concentrations ranged from 9.54 μg/ml (52.24 μM) in patients receiving 500 mg/m^2^ to 30.71 μg/ml (168.10 μM) in patients receiving 1500 mg/m^2^. The mean t_max_ occurred between 2.50 and 3.75 hours, in all dose groups. The mean AUC_0–6 h_ ranged from 39 hr-μg/ml at 500 mg/m^2^, to 121 hr-μg/ml in the 1500 mg/m^2^ dose group. The highest single serum concentration measured was 78.53 g/ml during cycle 1 in one patient in the highest dose group. This subject’s serum levels were otherwise unremarkable when compared with the other subjects in this dose group. As seen in Fig 3 and Table 3, there was significant variation in DFMO PK parameters among patients, possibly related to differences in dose administration time relative to sampling times, and the overall duration of sampling relative to the elimination half-life of DFMO, which is 2–4 hours (50) in adults. However, mean Cmax and AUC clearly increased in a linear fashion, in proportion to the oral doses administered, and mean tmax was consistent across dose groups.

**Table 3:** DFMO Pharmacokinetic Parameters (mean ± SD) by Dose Level.

PO BID Dose (mg/m^2^)	Cycle	C_max_ (mcg/ml) mean±SD	t_max_ hours	AUC_0–6 hrs_ (mcg/ml)●hrs
500	1	9.54 ± 5.36	3.75 ± 1.39	39.90 ± 24.16
750	1	11.93 ± 5.22	3.60 ± 1.26	47.36 ± 18.57
	2	14.23 ± 7.92	2.60 ± 0.89	62.84 ± 39.47
1000	1	14.71 ± 9.07	3.17 ± 1.60	60.05 ± 34.53
	2	14.33 ± 6.18	3.00 ± 0.00	50.18 ± 32.57
1500	1	28.89±14.96	2.88 ± 1.45	108.38 ± 53.23
	2	30.71 ± 8.18	2.50 ±0.90	120.69 ± 31.22

#### Rationale for genetic and metabolic markers of polyamine metabolism and pharmacodynamics (PD) measures of DFMO effect

**Fig 4:**
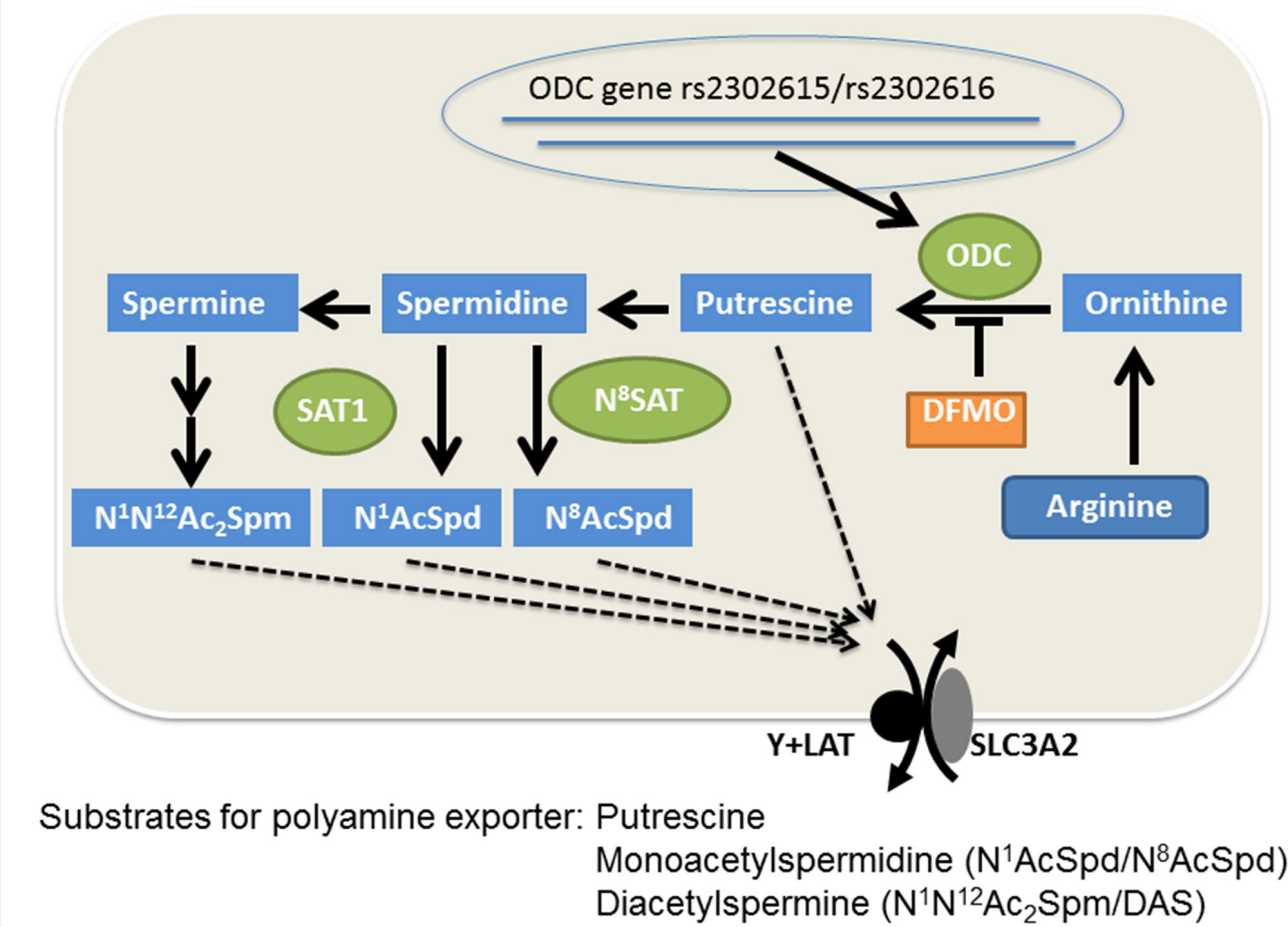
Rationale for DFMO- and specific genetic and metabolic markers of DFMO effect, in neuroblastoma . ODC transcription is influenced by specific genetic variability, including the SNPs rs2302615 [19, 22] and rs2302616 [24]. The DFMO target ODC decarboxylates ornithine to form the diamine putrescine, which is then metabolized into longer chain amines. Spermidine is a substrate for two acetyltransferases that monoacetylate this amine at either the N^**1**^ or N^**8**^ positions. Spermine is a substrate for one of these transferases (SAT1), which diacetylates this amine. Putrescine, the monoacetylspermidines and diacetylspermine are all substrates for the solute carrier transporter SLC3A2/Y+LAT, which exports these amines.

Fig 4 depicts the polyamine metabolic pathway and highlights the relationship between ODC genotypes (rs2302615 and rs2302616), affecting ODC expression, and their relationship to urinary polyamines. The figure shows the substrate relationships for the diamine and acetylpolyamine exporter [42–44], which include putrescine, monoacetylspermidine and diacetylspermine (DAS) but not spermidine or spermine. Levels of these exported amines might be expected to reflect changes in tissue ODC expression, as polyamine export is known as one component of polyamine homeostatic regulation [45].

First morning void spot urines from each patient were evaluated for polyamines as described in Methods. Table 4 shows data (means ± SD) at baseline (cycle 1, day 1) for seven metabolites in the polyamine pathway, including putrescine, spermidine, spermine and the acetylderivatives of spermidine and spermine. Listed in rank order in this table, N^8^AcSpd was the most prevalent amine in the urines of these patients at baseline, followed by N^1^AcSpd, putrescine, DAS, spermine, N^1^-acetylspermine (N^1^AcSpm) and spermidine. Values for each metabolite varied significantly, as indicated by the large standard deviation for each metabolite.

**Table 4:** Urinary polyamine metabolites from patients at baseline and during first two weeks of DFMO therapy.

Polyamine	C1D1 Mean (μmol/g Creatinine) (N = 19)	Standard Deviation (μmol/g Creatinine)	P-value for decrease from C1D1 to C1D8 (N = 19)*	P-value for decrease from C1D1 to C1D15 (N = 16)*
**N** ^**8**^ **AcSpd**	4.72	3.18	NS**	NS
**N** ^**1**^ **AcSpd**	3.96	3.18	0.018	0.005
**Putrescine**	1.93	7.02	NS	NS
**N** ^**1**^ **N** ^**12**^ **Ac**_**2**_ **Spm**	0.80	0.62	NS	NS
**Spermine**	0.55	1.71	NS	NS
**N** ^**1**^ **AcSpm**	0.33	0.74	NS	NS
**Spermidine**	0.26	0.25	NS	NS

C1D1 = cycle 1 day 1 (defined as baseline)

C1D8 = cycle 1 day 8 after starting DFMO on day1

C1D15 = cycle 1 day 15 after starting DFMO on day 1

*Determined by Friedman two-way analysis of variance

**Not significant

To determine if these baseline values were affected by treatment, all seven of these metabolites were evaluated for changes over the first two week period of treatment. Only N^1^AcSpd (N = 15 cases) showed a significant change over time (p = 0.004 unadjusted and p = 0.036 Bonferonni adjusted).

The changes in N^1^AcSpd were then further evaluated by paired comparisons between each of the 3 days (baseline versus day 8, baseline versus day 15, and day 8 versus day 15). The paired comparisons show that there was a significant decline from Day 1 to 8 (p = 0.018) for the N = 19 patients with Day 1 and 8 data, and a significant decline from Day 1 to 15 (p = 0.005) for N = 16 patients with Day 1 and 15 data. No change was seen between Day 8 and 15 (p = 1.000) for those N = 16 patients with complete data.

A standard repeated measures analysis of variance was used to confirm the apparent changes in N^1^AcSpd during the first two weeks of treatment. This parametric approach also used the 15 complete cases as did the paired comparisons analysis using the Friedman's test. The within subject results identify a significant linear effect (p = 0.003) and a marginal quadratic effect (p = 0.075). This analysis indicates that the mean values of N^1^AcSpd decline over time with most of the decline occurring during the first week of treatment. The bending or bottoming out at Day 8 and 15 leads to the quadratic effect. This is consistent with the paired Friedman's comparisons. As was the case with the overall Friedman's test, the overall change with the univariate repeated measures model show a significant change over time (p = 0.002).

We looked for patterns of these metabolites in relationship to ODC genotypes and the treatment period to determine if changes might be associated with either genetic factors or therapy. Table 5 lists individual patients rank-ordered by PFS and includes ODC genotype and urinary polyamine contents. For simplicity, in Table 5 we only show the sum of putrescine, N^1^AcSpd, N^8^AcSpd and DAS, which are true substrates for the tissue polyamine exporter. Table 6 presents results of associations of baseline and changes in urinary polyamines after one week of DFMO therapy and PFS with ODC genotypes. Median PFS was over 3 times greater in patients with any minor T allele, compared to GG, at rs2302616 (209 days compared to 62 days) with marginal statistical significance (P = 0.056). Differences in PFS by rs2302615 were not statistically significant. The variation observed in baseline urinary polyamines seemed to be at least partially explained by ODC genotype. Levels of urinary substrates for the polyamine exporter were nearly twice as high in samples from patients with the minor T-allele, compared to those with the GG genotype, at rs2302616 (P = 0.021). Urinary polyamines were higher for the GG genotype, compared to any A, at rs2302615, but this difference was not significant (P = 0.67). The effect of DFMO treatment was more pronounced as a function of ODC genotype. Urinary polyamine levels decreased by nearly 50% from baseline values after one week of DFMO therapy in patients with the minor T allele at rs2302616, while increasing nearly 25% in patients with the GG genotype at rs2302616 (P = 0.003). The effect of DFMO was also quantitatively greater in patients with the GG genotype, compared to any A allele, at rs2302615, but the difference was not statistically significant.

**Table 5:** Rank-ordered PFS by DFMO dose, ODC genotype and urinary polyamines.

Patient #	PFS (days)	Response after Cycle 1 (DFMO)	Best Response (CT/MIBG)	Status or Reason off study*	DFMO Dose (mg/m^2^)	ODC SNP rs2302615/ rs2302616	UPA **Cycle 1 Day 1 (μmol/g Creatinine)	UPA **Cycle 1 Day 8 (μmol/g Creatinine)	DAS increase from Cycle 1 Day 1
1	1573	SD	SD/PR	Alive (PF)	500	GA/TG	NA***	15.7	NA
2	1559	SD	SD	Alive (PF)	500	GG/TG	19.72	11.69	No
3	663	SD	SD/PR	Alive (PF)	1500	GG/TT	9	5.61	Yes
4	418	SD	SD	PD	750	GA/GG	2.25	7.8	No
5	239	SD	SD	PD	1000	GG/TG	40.12	8.71	No
6	209	PD	(CT Neg)/PR	PD	1500	GA/TG	12.23	3.98	No
7	136	PD	SD	2^nd^ Leukemia	1500	GG/GG	4.58	6.99	No
8	103	SD	SD	PD	750	GG/GG	5.04	NA***	No
9	94	SD	SD	PD	500	GG/TG	10.18	4.2	Yes
10	67	SD	PD	PD	750	GG/GG	26.75	22.08	Yes
11	64	PD	PD	PD	1000	AA/GG	4.97	3.89	Yes
12	62	SD	SD	PD	1500	AA/GG	2.85	3.77	No
13	62	SD	SD	PD	1500	GA/TG	11.53	8.81	Yes
14	62	SD	SD	PD	1500	GA/TG	15.35	7.38	Yes
15	59	PD	PD	PD	1000	GA/GG	6.8	3.28	Yes
16	57	SD	SD	PD	750	GG/GG	2.16	1.94	Yes
17	31	PD	PD	PD	750	GA/GG	15.34	13.46	Yes
18	21	PD	PD	PD	1500	GG/TG	7.49	5.93	Yes

*PF = progression free, PD = progressive disease

2^nd^ Leukemia = secondary leukemia

**Substrates for the tissue polyamine exporter SLC3A2 include the sum of putrescine, N1AcSpd, N8AcSpd and DAS; D1C1 = day 1, cycle 1, D8C1 = day 8 cycle 1

***NA = samples not available

**Table 6:** Association of ODC genotypes with polyamine markers and treatment responses.

ODC SNP	rs2302615			rs2302616		
Genotype	GG	Any A	P value	GG	Any T	P value
PFS	103 (67,NA)	62 (62,NA)	0.51	64 (59,NA)	209 (62,NA)	0.071
UPA C1D1	14.0 (7.9,24)	8.9 (5.4,12)	0.67	7.9 (4.2,15)	16 (11,27)	0.021
UPA (C1D1–C1D8)/ C1D1 X 100%	26 (-4.3,47)	-6.3 (-120,37)	0.88	-27 (-130,13)	47 (35,61)	0.0030

Urinary polyamines, especially DAS were also associated with disease progression. Urine samples were collected at intervals after baseline. For simplicity, Table 5 indicates whether DAS (or other urinary polyamine metabolites) increased from baseline values. Data was available from 17 of 18 patients evaluable for PFS. Baseline samples were not available for one patient in this group. Total urinary polyamines (putrescine+N^1^AcSpd+N^8^AcSpd+DAS) increased on average 6.56±16.29 μmol/g Creatinine from baseline in patients that experienced disease progression less than 100 days after start of therapy. Urinary polyamines decreased on average 1.57±3.37 μmol/g Creatinine in patients in whom disease progression occurred after 100 days from start of therapy. Urinary DAS increased in 9/10 patients with disease progression occurring within 100 days of therapy start, but in only 1/7 patients progression free up to 100 days (P = 0.056).

### Response

**Fig 5:**
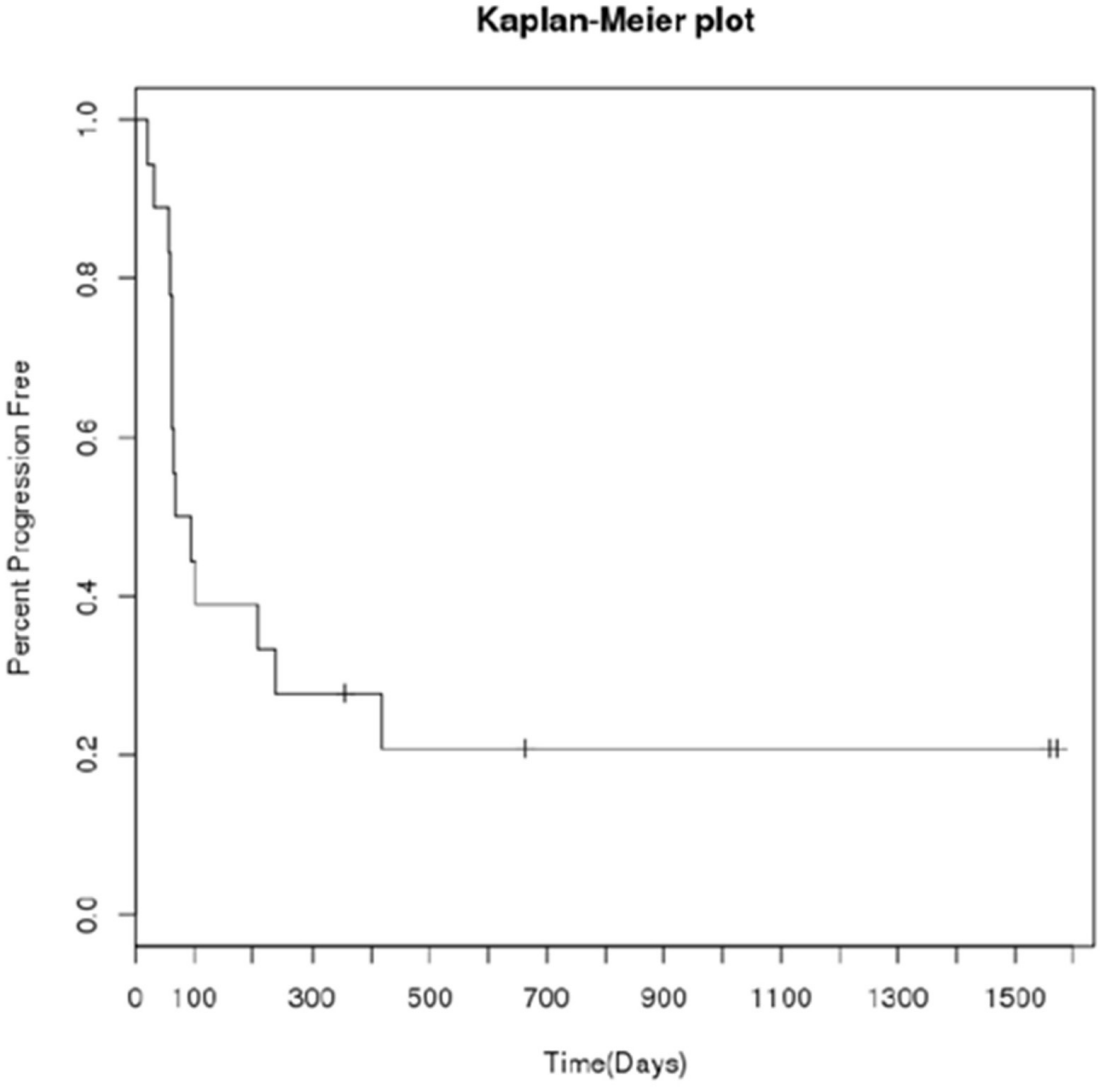
Progression free survival (PFS) and overall survival (OS) rates in patients enrolled in NMTRC 002 (N = 21). The number of patients shown at risk for disease progression (PFS) or death (OS) is shown in the figure.

While this was a phase I study and was not powered to evaluate anti-tumor efficacy, tumor response and clinical response were monitored. Eighteen subjects were evaluable for efficacy following treatment. The response for patients after one cycle of DFMO alone included 12 patients with stable disease and 6 patients with progressive disease. Overall response, after treatment with both DFMO and etoposide, utilizing Response Evaluation Criteria In Solid Tumors (RECIST) criteria, MIBG evaluation and bone marrow disease showed; 1 patient had a best response of PR (MIBG evaluable disease only), 12 subjects has a best response of stable disease by RECIST (with 2 of these subjects having PR on MIBG and one subject having CR in bone marrow) and 5 had a best response of progressive disease. Three subjects were evaluated by PET scans, of which two had a complete response of PET activity and one a partial response, though PET scans were not routinely performed on all subjects. These 3 patients are those that remain without progression on this study. It should be noted that response attribution may be related to the incorporation of etoposide for patients during cycle 2–5. A Kaplan- Meier plot of progression-free survival (PFS) is shown in Fig 5. The median progression free survival for all 18 evaluable subjects was 80.5 (95% CI: 62–418) days. Three patients remain alive without progression of disease between 2–4.5 years after starting DFMO.

## Discussion

This is the first clinical study of an oral dosing form of DFMO in any pediatric population. The recommended DFMO dose for Phase II studies is 500–1500 mg/m2 PO BID. The finding that this dose range is well tolerated by pediatric patients is notable. The results of this trial corroborate the safety of this agent noted in cancer chemoprevention studies in adults, where oral DFMO doses were 250–500 mg/m2 daily and ranged from 3–4 years in treatment duration [46, 47]. Doses used in this trial, similar to adult dosing, were chosen using metronomic dosing to attain biological activity as shown by the decrease in urinary polyamines and responses seen. Therefore we did not dose escalate to define a maximum tolerated dose. The concept of metronomic therapy, using low-dose daily medications to target disease, is often better tolerated than high dose chemotherapy as seen in this patient population. There is increased interest in this method for extensively pretreated patients in which quality of life is an important consideration [31, 48, 49].

DFMO is a highly targeted therapy that selectively inhibits ODC and acts to suppress the synthesis of putrescine and the polyamines. The rationale for DFMO in NB is based in part on its role as an inhibitor downstream of MYCN, which is a major risk factor in this disease [50, 51]. The results from this study suggest that genetic variability affecting ODC expression, specifically the rs2302616 SNP, is associated with increased polyamines, enhanced susceptibility to the ODC inhibitor DFMO and subsequent increased responsiveness to DFMO containing therapies in patients with NB. In this study response does not appear to be dependent on DFMO dose but rather correlates with genotype. These findings suggest that NBs in a subset of patients become “addicted” to polyamines in the manner suggested by Weinstein and colleagues for other cancer causing genes [52, 53], and as such become susceptible to therapies targeting this addiction.

In spite of these associations, some patients with the ODC risk allele did not respond to the DFMO plus etoposide therapy. It has been speculated that failures of therapies targeting single oncogenes are due to resistance mechanisms arising from acquisition of other activating mutations affecting additional signaling pathways [53]. Choi et al have recently reported evidence in support of this concept [54]. Their results suggest that DFMO combinations targeting other genetic features of NB [55–57] may be beneficial to patients not responding adequately to DFMO+/- etoposide.

The PK findings in this work demonstrate that DFMO dosing in children yields serum DFMO concentrations that are very similar to those reported in adult studies, as the concentration ranges overlap, for equivalent oral doses [58] [59]. The Tmax values observed in NB patients were also comparable to the values reported in adults [50]. The finding that clinical benefit was observed for a number of patients in this study, along with the reported efficacy of DFMO at these concentrations in adult cancer prevention studies, indicates that biologically effective doses of DFMO are in the 50–150 μM range. DFMO doses in this range do not kill NB cells [56], suggesting other mechanisms of DFMO action. One non-cytotoxic mechanism described recently is the suppression of metabolites involved in DNA synthesis [60]. Other non-cytotoxic mechanisms could involve inflammation [61] and/or immune responses [62]. A recent publication suggests the promotion of tumor immunity by polyamine blockade in a T cell dependent manner [63].

The secretion of polyamines in the urine as markers of neoplasia was proposed over 40 years ago [64]. Technology and limited understanding of the metabolism and transport of these polycationic molecules restricted their development. It is now appreciated that export of the polyamines is a highly regulated process, involving acetylation of spermidine and spermine, which enables them to act as counterions for a solute carrier transporter that facilitates arginine transport [42–44], as depicted in Fig 4. Substrates for the exporter of tissue polyamines has the general structure R_1_-NH_2_^+^-(CH_2_)_n>2_-NH_2_^+^-R_2_ [42]. Thus, putrescine, monoacetylspermidine and diacetylspermine, but neither spermidine nor spermine, are substrates for this exporter and might be expected to appear in the urine as a consequence of tissue attempts at homeostatic regulation under conditions of elevated polyamine metabolism. Spermidine, spermine and monoacetylspermine appear in the urine, but are likely either systemic degradation products resulting from cell lysis, serum amine oxidases (e.g. spermidine) or products of non-mammalian flora.

We assessed the relevance of seven polyamine metabolites in the urine, including those that are substrates for the polyamine exporter SLC3A2 and include putrescine, N^1^AcSpd and N^1^, N^12^Ac_2_Spm. Only N^1^AcSpd was affected in a statistically significant manner by DFMO treatment during the first few weeks of therapy. This species is one of the most prevalent polyamine metabolites in urine and is notable in that it is targeted for export by acetylation by the SAT1 gene product, which is physically linked to the SLC3A2 exporter [43]. SAT1 also associates with ODC to form a potential metabolic channel for putrescine and polyamine synthesis and export.

This is the first study to examine the polyamine titers in spot urine samples from NB patients. DFMO treatment reduced urinary N^1^AcSpd contents during the first two weeks of treatment in the population as a whole. Reductions in urinary polyamine levels were most significant in patients with the ODC minor T risk allele at rs2302616. Disease progression was associated with increases in urinary levels of especially DAS, although putrescine and monoacetylspermidine levels were elevated in some patients.

Diacetylspermine has previously been identified as a marker for tumor progression in adults with colon and breast cancer [34, 65]. Although the NMTRC 002 study was a small study of 21 patients, urinary levels of polyamines, especially DAS appear to fluctuate with disease state and may be a marker of disease state that can be evaluated during therapy. These associations are currently under investigation in other phase I and II trials in patients with DFMO in NB within the NMTRC. These increases could reflect mechanisms of resistance including elevated ODC enzyme levels requiring increased amounts of DFMO. It should be noted that no obvious DFMO dose dependent responses were observed for either reductions of urinary polyamines or increases in PFS responses in this study. Subsequent studies investigating DFMO dose escalation in more detail are in progress.

## Supporting Information

S1 Protocol Study protocol. (PDF)

S1 Table Enrollment Characteristics and Previous Relapse Therapies prior to Enrollment. (XLSX)

S1 TREND Checklist TREND checklist. (PDF)
