# Supplementary material for: A Phase I Trial of DFMO Targeting Polyamine Addiction in Patients with Relapsed/Refractory Neuroblastoma
Source: PLoS One. 2015 May 27;10(5):e0127246. doi: 10.1371/journal.pone.0127246 (PMC4446210; doi:10.1371/journal.pone.0127246)
Supplement: S1 Protocol — (PDF) [file pone.0127246.s001.pdf]

Protocol DFMO Phase I

NMTRC 002

A Phase I Trial for Refractory or Relapsed Neuroblastoma with DFMO alone and in combination with Etoposide.

Version 5.0 August 12, 2011

Contact Information

Study Chair:

Giselle Sholler, MD
NMTRC Chair
Van Andel Research Institute- 5th floor
333 Bostwick NE
Grand Rapids, MI 49503
Tel: (616) 234-5799
giselle.sholler@vai.org

Safety Officer:

Donald L. Durden MD PhD
Professor and Vice Chair for Research
Department of Pediatrics
Director Pediatric Oncology Research
Associate Director, Pediatric Oncology
Moores UCSD Cancer Center RM 3312
3855 Health Sciences Drive #0819
University of California, San Diego
La Jolla, CA 92093-0819
ddurden@ucsd.edu
PH: 858-534-3355
FAX: 858-822-0022

STUDY COMMITTEE

STUDY CHAIR:

Giselle Sholler, MD
NMTRC Chair
Van Andel Research Institute- 5th floor
333 Bostwick NE
Grand Rapids, MI 49503
Tel: 616-234-5799
giselle.sholler@vai.org

STUDY COMMITTEE MEMBERS:

Andre Bachmann, PhD
Cancer Research Center of Hawaii &
Dept. of Cell and Molecular Biology
John A. Burns School of Medicine
University of Hawaii at Manoa
1236 Lauhala Street
Honolulu, Hawaii 96813, USA
Phone office: ++808-586-2962
Fax.: ++808-586-2970
E-mail: abachmann@crch.hawaii.edu

William Roberts, MD
Pediatric Hematology/Oncology
Rady Children's Hospital San Diego
3020 Children's Way MC5035 San Diego, CA 92123-4282
Tel: (858) 966-5811
Fax: (858) 966-8035
Email: wroberts@chsd.org

William Ferguson, MD
Pediatric Hematology-Oncology
Cardinal Glennon Children's Medical Center
Saint Louis University School of Medicine
1465 S. Grand Blvd.
Saint Louis, MO 63104
Tel: (314) 577-5638
Fax: (314) 268-4081
Email: ferguws@slu.edu

Randy Wada, MD
U of Hawaii/Kapiolani Med Ctr for Women & Children
Molecular Carcinogenesis
1236 Lauhala Street
Honolulu, HI 96813
Tel: 808 586-2963
Fax: 808 586-2970
Email: tbear@crch.hawaii.edu

Genevieve Bergendahl, RN
Clinical Program Director
NMTRC
Van Andel Research Institute- 5th Floor
333 Bostwick NE
Grand Rapids, MI 49503

Tel: (616) 234-5799
E-Mail:
Genevieve.bergendahl@vai.org

Joel Kaplan, D.O.,M.P.H.
Pediatric Hematology/Oncology
Blumenthal Cancer Center
1025 Morehead Medical Drive, Suite 600
Charlotte, NC 28204

Phone--704-381-9900
Fax--704-381-9901
Pager--704-355-4088 #8553
E-Mail:
Joel.Kaplan@carolinashealthcare.org

Jacqueline M Kraveka, DO
Assistant Professor of Pediatrics
Medical University of South Carolina
Division of Pediatric Hematology-Oncology
135 Rutledge Avenue, Room 480
MSC 558
Charleston, SC 29425

Office: 843-792-2957
Pager: 843-792-2123
Fax: 843-792-8912
E-Mail: kravekjm@musc.edu

Deanna Mitchell, MD
Medical Director, Coagulation Disorders Program
Assistant Professor, Pediatrics
Michigan State University
Helen DeVos Children’s Hospital
100 Michigan NE
Grand Rapids, MI 49503

Phone: 616-391-2086
FAX: 616-391-8873
E-Mail:
Deanna.Mitchell@devoschildrens.org

Don Eslin, MD
MDACCO
Pediatric Hematology/Oncology
50 W. Sturtevant St.
Orlando, FL 32806

Office: 321-841-8588
E-Mail:
Don.Eslin@orlandohealth.com

Timothy Higgins, MD
Dept of Radiology
Fletcher Allen Health Care, Main Campus- Patrick 1
111 Colchester Avenue
Burlington, VT 05401

Tel: (802) 847-3592
Email:
timothy.higgins@vtmednet.org

Kathleen Neville, MD
Children's Mercy Hospitals and Clinics
Director, Experimental Therapeutics
in Pediatric Oncology Program
2401 Gillham Rd.
Kansas City, MO 64108
Tel: 816-234-3059
Fax: 816-855-1958
kaneville@cmh.edu

Suman Malempati, MD
Assistant Professor
Division of Pediatric Hematology/Oncology
Doernbecher Children's Hospital
Oregon Health & Science University
3181 SW Sam Jackson Park Rd., CDRC-P
Portland, OR 97239-3098
Tel: 503-494-1543
Fax: 503-494-0714
malempat@ohsu.edu

INVESTIGATOR SIGNATURE SHEET

I have read the attached protocol and agree that it contains all the necessary details for performing the study.

I will provide copies of the protocol and of the preclinical and clinical information on the test article, which was furnished to me by the Neuroblastoma and Medulloblastoma Translational Research Consortium (NMTRC), to all members of the study team responsible to me who participate in the study. I will discuss this material with them to assure that they are fully informed regarding the test article and the conduct of the study.

Once the protocol has been approved by the Institutional Review Board (IRB)/Independent Ethics Committee (IEC), I will not modify this protocol without obtaining the prior approval of the NMTRC and of the IRB/IEC. I will submit the protocol modifications and/or any informed consent form (ICF) modifications to the NMTRC and the IRB/IEC, and approval will be obtained before any modifications are implemented.

I understand the protocol and will work according to it, the principles of Good Clinical Practice (GCP) [current International Conference of Harmonization (ICH) guidelines], and the Declaration of Helsinki (1964) including all amendments up to and including the Scotland revision (2000) and notes of clarification added in 2002 and 2004.

____ ______________

Investigator's Signature Date

____

Investigator’s Printed Name

____

Investigational Site Name

TABLE OF CONTENTS

CONTACT INFORMATION 2

STUDY COMMITTEE 3

INVESTIGATOR SIGNATURE SHEET 6

TABLE OF CONTENTS 7

PROCEDURES AND ASSESSMENTS 13

1.0 PROTOCOL CONCEPT 14

2.0 BACKGROUND AND PRELIMINARY DATA 14
- 2.1 PRECLINICAL WORK 14
- 2.2 CLINICAL WORK 20

3.0 STUDY OBJECTIVES 22
- 3.1 PRIMARY OBJECTIVE: 22
- 3.2 SECONDARY OBJECTIVES: 22

4.0 STUDY DESIGN 22
- 4.1 CYCLE 1 : DFMO 22
- 4.2 COMBINATION CYCLES (CYCLES 2-5): DFMO WITH ETOPOSIDE. 23
- 4.3 RESPONSE EVALUATIONS 23
- 4.4 PHARMACOKINETICS 23
- 4.5 BIOLOGICAL STUDIES 23

5.0 PATIENT SELECTION 24
- 5.1 INCLUSION CRITERIA: 24
- 5.2 EXCLUSION CRITERIA: 24

6.0 STUDY DRUG ADMINISTRATION 26
- 6.1 DFMO 26
- 6.2 ETOPOSIDE 30
- 6.3 DOSE LIMITING TOXICITIES AND GUIDELINES FOR DOSE MODIFICATIONS 32
- 6.4 GUIDELINES FOR SUBSEQUENT CYCLES 32

7.0 STUDY PROCEDURES AND ASSESSMENTS....34
7.1 ENROLLMENT OF PATIENTS....34
7.2 SCREENING....35
7.3 TREATMENT PHASE – CYCLE 1 36
7.4TREATMENT PHASE – CYCLE 2 37
7.5 TREATMENT PHASE – CYCLE 3 38
7.6 TREATMENT PHASE – CYCLE 4 39
7.7 TREATMENT PHASE – CYCLE 5 40
7.8 SUBSEQUENT CYCLES 41
7.9 OTHER SCHEDULES: 41
7.10 STUDY COMPLETION 42
7.11 FOLLOW-UP VISIT 42
7.12 SURVIVAL FOLLOW-UP 42

8.0 PHARMACOKINETIC AND URINE STUDIES 43

9.0 RESPONSE CRITERIA....44

10.0 ADVERSE EVENT REPORTING 48
10.1 DEFINITIONS 48
10.2 GRADING AND RELATEDNESS OF ADVERSE EVENTS 50
11.0 PATIENT WITHDRAWAL AND TRIAL DISCONTINUATION 51
11.1 CRITERIA FOR PATIENT WITHDRAWAL 51
11.2 PATIENT REPLACEMENT....51
11.3 TRIAL DISCONTINUATION 51

12.0 DATA ANALYSIS....52
12.1 DATA QUALITY ASSURANCE....52
12.2 STATISTICAL ANALYSIS 52

13.0 ADMINISTRATIVE PROCEDURES....53
13.1 PATIENT INFORMED CONSENT 53
13.2 ETHICAL CONDUCT OF THE STUDY AND IRB/IEC APPROVAL....53
13.3 DATA SAFETY MONITORING BOARD (DSMB) 54
13.4 MONITORING....54
13.5 PRE-STUDY DOCUMENTATION....54
13.6 CONFIDENTIALITY....55
13.7 SOURCE DOCUMENTS....55
13.8 RECORD RETENTION 55

14.0 BIOLOGICAL EVALUATION 56

APPENDIX I: PERFORMANCE STATUS/SCORES....59

A Phase I Trial for Refractory or Relapsed Neuroblastoma with DFMO alone and in combination with Etoposide.

PROTOCOL SYNOPSIS

**PROTOCOL TITLE**	A Phase 1 Trial of DFMO as a Single Agent and in Combination with Etoposide in Patients with Refractory or Recurrent Neuroblastoma
**PROTOCOL NUMBER**	NMTRC 002
**PHASE OF DEVELOPMENT**	Phase 1
**OBJECTIVES**	**Primary**<br>To determine the safety, tolerability and maximum tolerated dose (MTD) of DFMO as a single agent and in combination with etoposide in pediatric and young adult patients with refractory or recurrent neuroblastoma<br>**Secondary**<br>• To evaluate the activity of DFMO as a single agent and in combination with etoposide in these tumor types based on:<br>♦ Progression free survival (PFS)<br>♦ Overall response rate (ORR)<br>• To evaluate the pharmacokinetics (PK) of DFMO as single agent<br>• Biology studies to include: effect on polyamine depletion, ODC activity, genomic analysis of cells pre- and post-treatment, correlation of *in vitro* response to *in vivo* response, flow cytometry of tumor burden in bone marrow and biomarker development
**STUDY DESIGN**	DFMO is an open label, multicenter, dose escalation study in patients with refractory or recurrent neuroblastoma.<br>**Cycle 1: DFMO**<br>This study uses a standard 3+3 design in which cohorts of 3 patients will receive one cycle of oral DFMO at a starting dose of 500 mg/m^2 BID of a 21-day cycle. The dose escalation scheme for subsequent cohorts and modifications for dose limiting toxicities (DLT) are detailed in the protocol. The MTD of single agent therapy will be defined as the dose level below which DLTs are seen in ≥ two of six patients dosed.<br>**Cycle 2-5: Combination Cycles: DFMO and Etoposide**<br>Each patient will receive etoposide in addition to DFMO at the same dose that was well tolerated in Cycle 1 for all remaining cycles. Dose and other modifications of the combination therapy phase are detailed in the protocol.<br>Plasma concentrations of DFMO will also be determined at designated time points.
**ELIGIBILITY**	**Inclusion Criteria**<br>1. Age: 0-21 years at the time of diagnosis.<br>2. Diagnosis: Histologic verification at either the time of

original diagnosis or relapse of neuroblastoma

3. Disease Status: Refractory or relapsed neuroblastoma
4. Measurable or evaluable disease, including at least one of the following: Measureable tumor >10mm by CT or MRI; A positive MIBG and abnormal urinary catecholamine levels; Positive bone marrow biopsy/aspirate.
5. Current disease state must be one for which there is currently no known curative therapy
6. A negative urine pregnancy test is required for female subjects of child bearing potential (onset of menses or ≥13 years of age).
7. Patients without bone marrow metastases must have an ANC > 500/µl and platelet count >50,000/µl
8. Organ Function Requirements
   - Subjects must have adequate liver function as defined by AST and ALT <10x upper limit of normal
   - Serum bilirubin must be ≤ 2.0 mg/dl
   - Serum creatinine must be ≤ 3 mg/dl x upper limit of normal
9. Informed Consent: All subjects and/or legal guardians must sign informed written consent. Assent, when appropriate, will be obtained according to institutional guidelines

Exclusion Criteria

1. Life expectancy <2 months or Lansky score <30%.
2. Investigational Drugs: Subjects who are currently receiving another investigational drug are excluded from participation.
3. Anti-cancer Agents: Subjects who are currently receiving other anticancer agents are not eligible. Subjects must have fully recovered from the effects of prior chemotherapy (hematological and bone marrow suppression effects).
4. Infection: Subjects who have an uncontrolled infection are not eligible until the infection is judged to be well controlled in the opinion of the investigator.
5. Subjects who, in the opinion of the investigator, may not be able to comply with the safety monitoring requirements of the study, or in whom compliance is likely to be suboptimal, should be excluded.

Additional criteria:

Patients willing to participate in the correlative biologic studies will sign an additional consent form to provide tumor tissue for analysis of polyamine content, ODC activity, MYCN levels and protein and gene expression.

<table>
<tr><td>ESTIMATED NUMBER OF PATIENTS/GEOGRAPHIC REGIONS</td><td>At least three and up to six patients per cohort, and an estimated four cohorts (based on safety) may be enrolled for a projected total of 12 to 24 patients. Up to 10 sites in the United States will participate.</td></tr>
<tr><td>LENGTH OF STUDY</td><td>Accrual to this study is estimated to be at least 1.5 years, with follow up for one year after last patient completion.</td></tr>
<tr><td>INVESTIGATIONAL PRODUCT DOSE/ROUTE/REGIMEN</td><td>DFMO is an oral agent that will be administered daily on a 21-day cycle. Starting dose and dose escalation with DFMO will proceed as follows in the absence of DLT:

Single Agent Therapy – Cycle 1: DFMO

<table>
<tr><th>DFMO Dose Level</th><th>Dose</th></tr>
<tr><td>1</td><td>500 mg/m^2 BID</td></tr>
<tr><td>2</td><td>750 mg/m^2 BID</td></tr>
<tr><td>3</td><td>1000 mg/m^2 BID</td></tr>
<tr><td>4</td><td>1500 mg/m^2 BID</td></tr>
</table>

Combination Therapy: DFMO and Etoposide
DFMO will be administered concurrently with etoposide in Cycles 2-5.</td></tr>
<tr><td>STUDY ASSESSMENTS</td><td>Refer to Table of Assessments for timing of study procedures.</td></tr>
<tr><td>CRITERIA FOR EVALUATION</td><td>Safety Measures: Primary objectives

• Safety analysis will be conducted on all patients who have received at least one dose of study drug, and will include the frequency of all adverse events and laboratory abnormalities as well as frequency of dose interruptions, dose reductions and treatment discontinuation

Response Measures: Secondary objectives
• Response:
♦ To determine the overall response rate (ORR) by the presence of radiologically assessable disease by cross-sectional imaging and in neuroblastoma by MIBG scans. Response will be defined by criteria in section 9.0; or disappearance of abnormal MIBG foci.
♦ Duration of response, defined as the period of time from when measurement criteria are met for complete response (CR) or partial response (PR), whichever is first recorded, until the first date that recurrent or progressive disease (PD) is objectively documented (taking as reference for PD the smallest</td></tr>
</table>

	measurements recorded since the treatment started)<br>♦ The assessment of response will include the initial measurable targets and will be performed after the first and second cycle, then after every other cycle. Serial results of bone marrow aspirates, trephine biopsies and urinary catecholamines will be reviewed for responding patients to confirm response or lack of progression.<br>♦ A patient will be defined as a responder if CR/PR is observed at any time of the treatment and confirmed no less than 1 month later<br>• Time to progression, defined as the period from the start of the treatment until the criteria for progression are met taking as reference the screening measurements<br>• Survival will be recorded at all times during the study, and follow-up will be every month for three months, then every three months up to one year.
**STATISTICS/SAMPLE SIZE ESTIMATE**	**Statistics**:<br>All baseline patient characteristics will be summarized in a tabular format. Safety data will be described for all patients receiving at least one dose of DFMO. Safety data will include values for hematology, serum chemistry, vital signs, and adverse events. The proportion of patients experiencing adverse events, serious adverse events, dose limiting toxicities and treatment delays will be summarized for each dosing cohort.<br><br>The proportion of patients experiencing progressive disease, stable disease, partial responses or complete responses will be summarized in tabular format. Progression free survival and duration of any responses will also be summarized.<br><br>The DFMO plasma concentration-time data will be determined for each patient as a single agent. These analyses will also be performed for metabolites of both drugs. The following PK parameters will be determined for DFMO:<br>• Cmax<br>• AUC (0 – 24 hr)<br>• Plasma half-life (t1/2)<br>• Plasma clearance (Cl)<br>• Vd<br>**Sample Size:**<br>There is no data on DFMO in the pediatric or adolescent population. As this is not a hypothesis testing study, no formal sample size calculations will be made.

Procedures and Assessments

	Pre	Cycles 1				Cycles 2-5				Subsequent Cycles	Follow Up
		Day 1	Day 8	Day 15	End of cycle	Day 1	Day 8	Day 15	End of Cycle		
Informed consent	X										
Medical & surgical history, demographics, histologic evidence of malignancy	X										
Prior therapy for malignancy	X										
Tumor Tissue (Pharmacogenomics)[a]	X										
Physical examination	X	X	X	X		X		X		X	X
Vital signs	X	X	X	X		X	X	X		X	X
ECOG performance/Lansky play status	X					X				X	
CBC and differential	X		X	X		X	X	X		X	X
Serum chemistries	X		X	X		X	X	X		X	X
Pregnancy test	X					X					
MIBG/CT/MRI Scan as needed	X				X				X*	X	X
Concomitant medications	X	X	X	X		X	X	X		X	X
BSA calculation		X				X				X	
Administration of DFMO		➔	➔	➔	➔	➔	➔	➔		➔	
Administration of Etoposide						➔	➔	Stop		➔	
Pharmacokinetics[c]		X	X			X	X				
Dispense (and collect) drug dosing diary		X				X					X
AE monitoring		X	X	X		X	X	X		X	X
Progression free Survival						X				X	X
Urine Polyamines		X	X	X		X					
Audiogram	X				X				X*		X[b]
Urine VMA/HVA		X				X					X[b]
Bone Marrow^	X					X*					X

a *Voluntary – additional informed consent required*
b end of cycle 5 and as clinically indicated
c Cycle 1 and Cycle 2 on days 1 and 8 at hour 0 (pre-dose), 30 minutes, 1 hour, 3 hours, and 6 hours
** at end of cycle 3 and 5, then every two cycles*
^Separate consent required

1.0 Protocol Concept

There is currently no curative treatment for children with relapsed/refractory neuroblastoma, and for these children the 5-year survival rate is <10%. As such, new therapeutic approaches are needed to treat these children.

These more aggressive forms of NB respond poorly to hormonal and chemotherapeutic approaches, and therefore, there is a great need for antineoplastic agents with novel mechanisms of action. The MYCN protein up-regulates *ornithine decarboxylase* (*ODC*), a gene encoding for the ODC enzyme that is pivotal in polyamine biosynthesis. High polyamine content and elevated ODC activities are commonly found in many tumors, and therefore, suppression of polyamines in cancer cells is an effective means to reduce tumor cell proliferation. Specific polyamine inhibitors such as DFMO and SAM486A have been evaluated in adult clinical cancer trials and DFMO will be evaluated in this pediatric NB trial as a standard 3+3 Phase I dose escalation trial.

2.0 Background and Preliminary Data

2.1 Preclinical Work

Neuroblastoma (NB) is a tumor of the autonomous nervous system originating from the adrenal medulla and autonomous ganglia in the chest and abdomen. After leukemia and cerebral tumors, NB is the third most frequent malignant tumor of childhood. The incidence in the United States is approximately one in 7,000 children (1). The therapy of NB is very difficult, especially in advanced stages of the disease with widespread metastases to liver, bone, lymph nodes, and bone marrow. Current therapy includes chemotherapy, radiation, and high dose chemotherapy with subsequent bone marrow transplantation. More recently, immunotherapy has been added using monoclonal antibodies to the GD_2 glycolipid antigen that is heavily expressed by NB cells (2, 3). Over the last 30 years, significant therapeutic progress has been made with an increase in the five-year relative survival rate from approximately 25% to 55%. However, almost 50% of patients are estimated to die of their tumor, and over the past decade or two, no improvement in the five-year survival rate of NB patients has been made (4). NB has a particularly poor prognosis in patients older than 2 years at diagnosis, advanced stage disease and/or disease characterized by *MYCN* gene amplification (5, 6). These more aggressive forms of NB respond poorly to chemotherapeutic approaches, and therefore, there is great need for a better understanding of the cellular regulation of *MYCN*-amplified NB tumors in an effort to search for alternative molecular drug targets. Although a role for the MYCN oncoprotein has been established in NB pathogenesis, the mechanism by which MYCN contributes to both the development of this disease and its poor prognosis is still unclear. The MYCN oncoprotein functions as a transcriptional regulator (7) and thus may influence tumorigenesis and patient survival by regulating the expression of key genes involved in the NB malignant phenotype. MYCN regulates the expression of genes that encode ornithine decarboxylase (ODC), the multi drug resistance-associated protein 1 (MRP1), and MDM2 (8). ODC is the rate-limiting enzyme in the production of polyamines (9). Although polyamines, and therefore ODC, are essential for normal cell proliferation, increased ODC activity can induce cellular transformation *in vitro* (10), and high ODC levels are associated with a variety of tumors,

including those of the brain and prostate (11, 12). Several studies have identified *ODC* as a target gene for the MYCN oncoprotein (7, 13, 14), and it is possible that ODC, and therefore polyamines, play a significant role in NB tumorigenesis.

Role of polyamines in cancer

The identification of novel inhibitors of enzymes involved in polyamine biosynthesis with antitumor activities has recently revived interest in polyamine homeostasis and in designing strategies of cancer chemotherapy (15-17). Selective pharmacological interference with the synthesis of natural polyamines results in tumor cell growth inhibition under both in vitro and in vivo conditions (15, 18). Although the precise mechanism, by which polyamines contribute to cell proliferation is not well known, it has been suggested that it may be a result of their ability to bind DNA and affect gene expression by bringing about structural changes in chromatin, thereby stimulating cell growth (19). Furthermore, the dramatic increases in the activity of ODC in certain tumor cells have been linked to G_1-S transition (20-22). As mentioned above, an apparent molecular basis for this derives from the fact that ODC is among those genes, which can be regulated by c-Myc and MYCN (13, 14, 23-25), both of which regulate entry into and exit from the cell cycle. Because cell growth is absolutely dependent on polyamines, interference with polyamine biosynthesis has long been considered a promising therapeutic approach against proliferative diseases, including various malignancies (10, 18, 26). α-difluoromethylornithine (DFMO or eflornithine), a suicide substrate inhibitor of ODC (27, 28), has been the prototype tool for the study of therapeutic effectiveness of polyamine depletion in experimental tumors (18, 29) (Fig. 1).

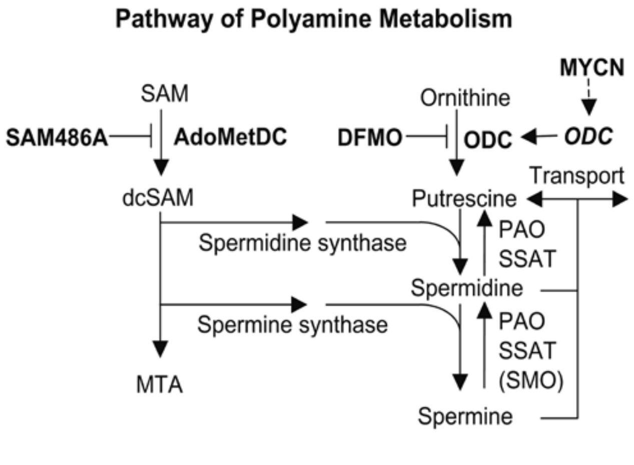

Figure 1. Pathway of polyamine metabolism showing two target enzymes of the polyamine inhibitors DFMO and SAM486A. By inhibiting ODC, DFMO depletes putrescine (Put) and spermidine (Spd) pools, whereas it only modestly affects spermine (Spm) pools. By inhibiting AdoMetDC, SAM486A depletes Spd and Spm, whereas it markedly increases Put. The inhibitor combination lowers all three polyamine pools until cells stop growing and the pools are no longer diluted by cell division. SSAT and PAO work in concert to acetylate and oxidize polyamines during retro-conversion. SMO converts Spm back to Spd without the need for an acetylation step. Most unfavorable NB tumors are associated with high levels of MYCN, a transcription factor that can upregulate *ODC* gene expression. AdoMetDC, *S*-adenosylmethionine decarboxylase; MTA, methyl-thioadenosine; ODC, ornithine decarboxylase; PAO, polyamine oxidase; SAM, *S*-adenosylmethionine; SMO, spermine oxidase; SSAT, spermidine/spermine N^1-acetyltransferase

DFMO inhibits cell growth of many cancer cells including NB and induces cell differentiation (30, 31). These processes are accompanied by an apparent depletion of putrescine (Put) and spermidine (Spd) pools (18, 26, 32). DFMO has also been shown to

induce apoptosis and inhibit metastasis in a human gastric cancer model (33). Although ODC has generally been considered as the enzyme catalyzing the rate-limiting step in polyamine biosynthesis, it has been shown that the supply of decarboxylated S-adenosylmethionine represents a second rate-limiting factor in polyamine biosynthesis (32). Thus, the enzyme S-adenosylmethionine decarboxylase (AdoMetDC) represents a second rational target for anti-cancer agent development (Fig. 1). Since the two enzymes are co-regulated by intracellular polyamine pools so that inhibition of one results in a compensatory increase in the other, it follows that targeted interference with a drug cocktail composed of ODC and AdoMetDC inhibitors (e.g. SAM486A) could be especially useful and is likely to sharpen the antiproliferative effects by causing complete depletion of the polyamine pools. Both DFMO and SAM486A have been independently tested in human Phase III and Phase II clinical trials, respectively (34-42), however, the combination of both inhibitors has not been assessed in clinical trials thus far.

The regulation of polyamines is further controlled by the enzymes spermidine/spermine N^1-acetyltransferase (SSAT) and polyamine oxidase (PAO) (Fig. 1). SSAT uses acetyl-CoA to form N^1-acetylspermidine and spermine (Spm) and the FAD-dependent PAO further retro-converts the N^1-acetyl derivatives to Spd and Spm. Each oxidation generates H_2O_2, which is an inducer of SSAT, thereby perpetuating the cycling. High local concentrations of H_2O_2 can lead to oxidative stress and cell death (43). Spermine oxidase (SMO) was recently identified and allows an alternative retro-conversion of Spm to Spd without the need of an acetylation step (44, 45).Finally, polyamines are also controlled by cellular transport mechanisms. While in most cases, the major polyamines exported from the cell are N^1-acetylspermidine or Put all three polyamines (Put, Spd, and Spm) are imported either by one or two types of carrier.

Role of polyamines in NB cell differentiation

The fluctuation in the levels of intracellular polyamines such as Put, Spd, and Spm has been observed in association with cell differentiation (46-48), and inhibition of ODC by DFMO and reduction in polyamine pools stimulates various cancer cells including NB cells to differentiate (31,49,50). DFMO treatment of NB cells can change the triangular NB morphology by inducing a different phenotype one which resembles elongated fibroblast-like cells without typical neuritic processes. By comparison, treatment with retinoic acid (RA) induces neural differentiation of NB cells as indicated by the outgrowth of definite neurites (39,50,51). The role of polyamines in cell differentiation has been studied for many years, and yet the precise role of polyamines at the cellular and molecular level is still not well understood and may play a key role in tumor regression.

Effect of polyamine inhibitors DFMO and SAM486A cell proliferation and apoptosis in NB cells

In our NB cell culture-based studies, we showed that pharmacological inhibition of the polyamine biosynthetic enzymes ODC and AdoMetDC with alpha-difluoromethylornithine (DFMO) and SAM486A, respectively, inhibits the proliferation of NB cells (LAN-1, NMB-7, and SK-N-SH) (52).

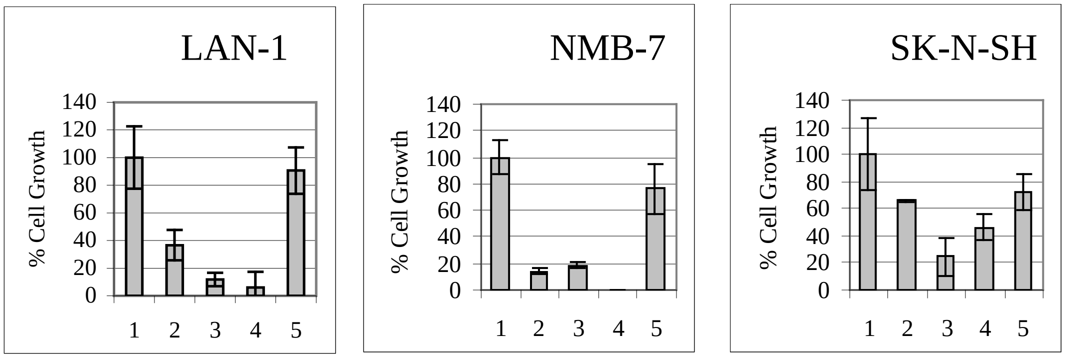

Figure 2. Effects of polyamine inhibitors DFMO and SAM486A on the proliferation of human NB cell lines LAN-1, NMB-7, and SK-N-SH. Cells were grown in the absence (*1*) or presence (*2-5*) of 5 mM DFMO (*2*), 10 μM SAM486A (*3*), 5 mM DFMO plus 10 μM SAM486A (*4*), and 5 mM DFMO plus 10 μM SAM486A, supplemented with 10 μM Spd (*5*) for 3 days and counted using a hemocytometer and trypan blue for exclusion of dead cells. Cell numbers were expressed as percentage (%) of cell growth assuming 100% cell growth for untreated control cells. Each sample was measured in duplicate and the data represent the mean of three separate determinations; bars ± SD. Representative light micrographs for LAN-1 and NMB-7 cells are shown in (70).

In LAN-1 and NMB-7 cells (both lines MYCN-amplified and p53 mutant), the observed inhibition was due to p27/Rb-mediated G_1 cell cycle arrest in response to DFMO and DFMO/SAM486A (but not SAM486A alone) treatment. Most intriguingly, DFMO alone and combined with SAM486A nearly abolished MYCN protein levels in treated NB cells (Fig. 3) (52). These results clearly demonstrate that the depletion of polyamines with ODC and/or AdoMetDC inhibitors rapidly reduces NB tumor cell proliferation.

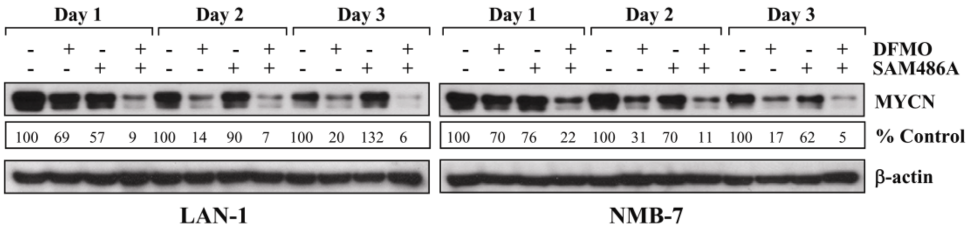

Figure 3. Western blot analyses of inhibitor-treated human NB cells. Effect on (**A**) MYCN in LAN-1 and NMB-7 cells treated with 5 mM DFMO, 10 μM SAM486A or the combination of both inhibitors. Cells were analyzed on days 1, 2, and 3. Equal amounts of total protein were loaded in all experiments. MYCN bands were quantified and normalized relative to β-actin. Values are presented as percentage (%) of controls of day 1, 2 or 3. (**c**). Data are representative of at least three independent experiments (n=3). (70).

Further investigation in *MYCN*-amplified, p53 mutant NB cells revealed that ODC inhibition by DFMO, in addition to inducing p27Kip1-mediated cell cycle arrest, also

activates an opposing signaling pathway via phosphorylation of Akt/PKB (53), thus possibly explaining the moderate efficacy of DFMO monotherapy in adult cancer clinical trials. These results clearly indicate the need for combination therapies, for example, using PI3K or Akt/PKB-targeted inhibitors to counter-act the opposing effect of Akt/PKB activation by DFMO.

Effect of polyamine inhibitor DFMO in a transgenic neuroblastoma animal model

Encouraging data by two groups (54, 55) recently emerged and confirmed the effect of DFMO *in vivo* using the *TH-MYCN* NB mouse model, and DFMO in combination with cisplatin and cyclophosphamide increased the tumor-free survival of TH-MYCN homozygous mice (Fig. 4) (54). Additional studies have revealed that DFMO combined with SAM486A act synergistically and result in a significantly reduced tumor burden in TH-MYCN mice (56).

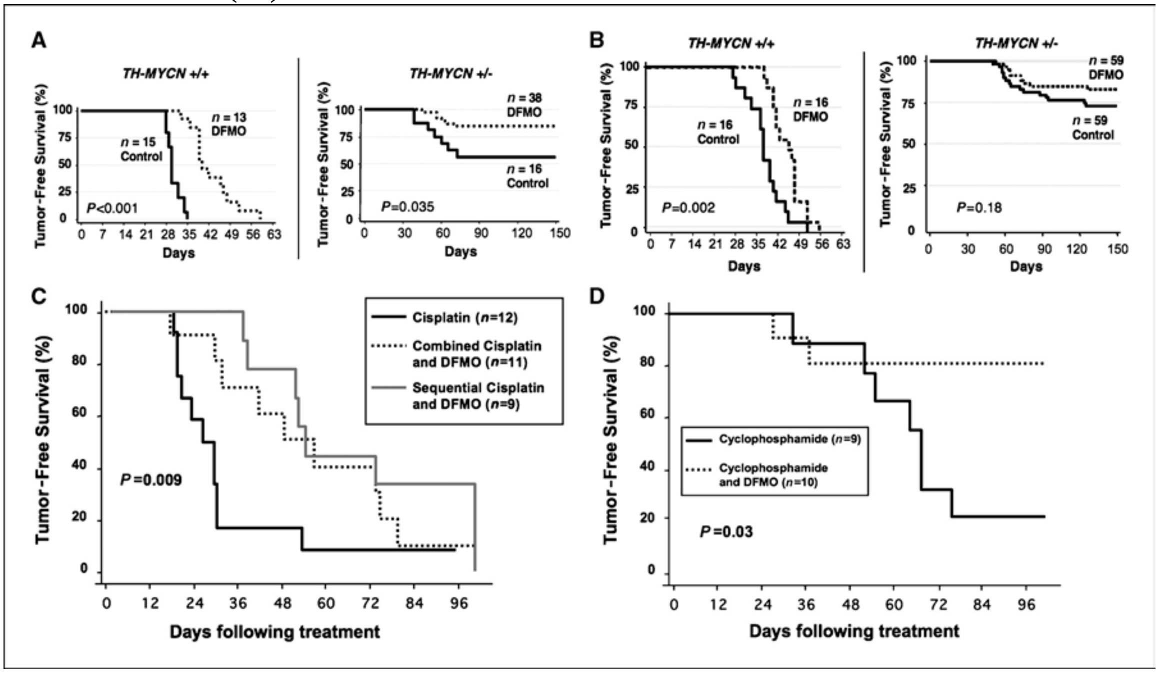

Figure 4. Extended tumor-free survival in neuroblastoma-prone mice treated with DFMO. **A,** tumor-free survival curves for homozygous (*TH-MYCN* +/+) or hemizygous (*TH-MYCN* +/-) mice stratified by DFMO therapy. DFMO-treated mice (dashed lines) received DFMO from birth onward (preemptive treatment trial). DFMO therapy was stopped at day 70 in tumor-free mice. **B,** delayed treatment trial: *TH-MYCN* homozygous and hemizygous mice were randomized to DFMO (dashed lines) or control (solid lines) following weaning at day 25. Tumor-free survival for *TH-MYCN* homozygous mice with advanced tumor from the time of treatment with (**C**) cisplatin alone (black line), cisplatin followed by DFMO (gray line), or cisplatin administered simultaneously with DFMO (dashed line) or (**D**) cyclophosphamide alone (solid line) or combined with DFMO (dashed line). P values using the method of Kaplan-Meier are shown (75).

Relevance of ODC in patients with neuroblastoma

Further evidence of the importance of ODC in NB tumorigenesis is available from our recent studies with human NB tumors. We analyzed the expression levels of ODC mRNA from 88 NB patients and found significant correlations between ODC expression and the overall survival probability. High levels of ODC were predictive of low survival probability and vice versa (Fig. 5A). Most surprisingly, ODC was also predictive in

tumors without MYCN amplification (Fig. 5B), thus suggesting that ODC also plays a role in NB tumorigenesis independent of MYCN amplification (57). These findings were independently confirmed by two other groups (54,55).

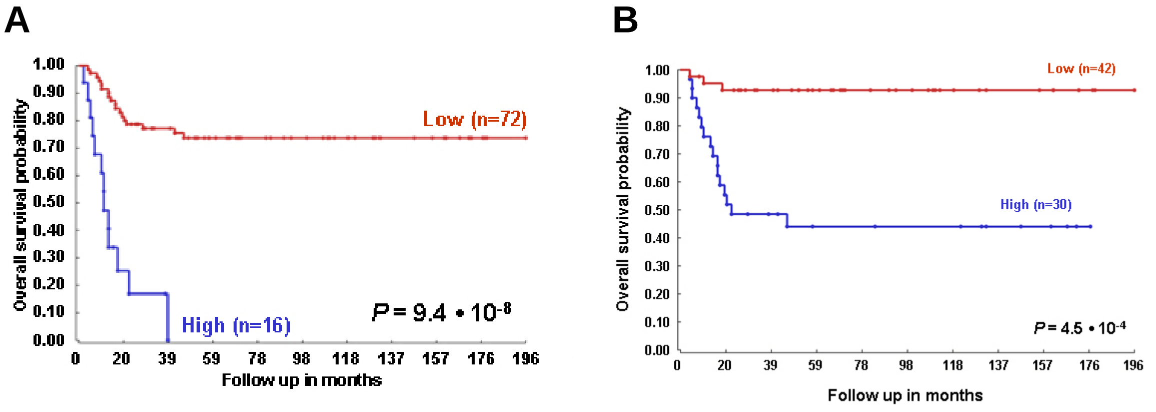

Figure 5. Correlation of ODC gene expression with NB patient survival prognosis. **A,** Kaplan-Meier graphs representing the survival prognosis of 88 NB patients based on high or low expression levels of ODC. Survival probability of NB patients (follow-up over 196 months) with low ODC expression is significantly higher than of patients with high ODC expression. **B,** Kaplan-Meier graphs representing the survival prognosis based on high or low expression levels of ODC stratified for patients without *MYCN* amplification. The survival probability of NB patients with low ODC expression is significantly higher than of patients with high ODC expression (80)..

Synergistic effects of DFMO and Etoposide

A strong rationale for the combination of DFMO with etoposide was provided by Dr. Gerner's team at the University of Arizona (58). His group showed that the anticancer agent etoposide (VP-16), which produces DNA strand scission in tumor cells, combined with DFMO results in synergistic cytotoxic effects in leukemic and myeloma tumor cells and also in mice bearing L1210 lymphocytic leukemia. Since etoposide in low dose therapy has been shown effective in neuroblastoma this combination was studied in our lab. A cell viability assay was performed on SMS-KCNR neuroblastoma cells treated with either vehicle, 25nM etoposide, 20mM DFMO, or a combination of 25nM etoposide and 20mM DFMO. The drug treatment duration was 48 hours. The results show that DFMO and etoposide in combination is more effective at killing neuroblastoma cells than either drug alone.

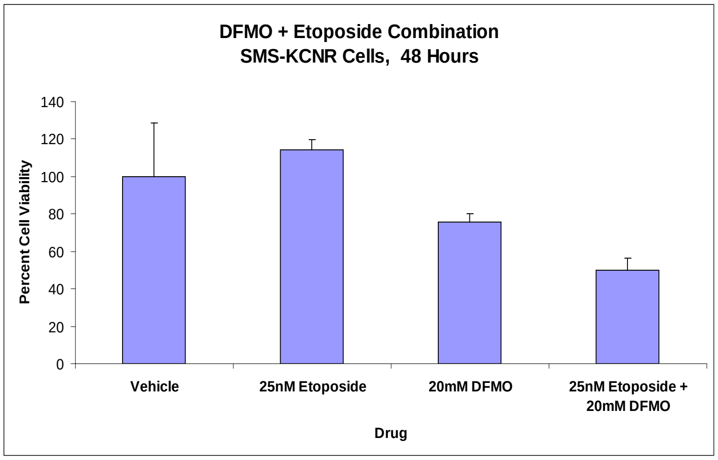

Figure 6. A Calcien AM viability assay was performed on SMS-KCNR neuroblastoma cells treated with 1) Vehicle, 2) 25nM Etoposide, 3) 20mM DFMO, or 4) a combination of 25nM Etoposide and 20mM DFMO.

2.2 Clinical Work

Chemopreventive effects of DFMO and sulindac in sporadic colorectal adenomas

A recent randomized placebo-controlled double-blind trial was performed by Drs. Meyskens and Gerner to test whether the combination of a low dose of DFMO plus a low dose of sulindac reduces the recurrence of human colorectal adenomas (59). This study revealed that recurrent adenomatous polyps in patients can be markedly reduced by a combination of low oral doses of DFMO and sulindac (Table 1). Moreover, the study provided strong evidence that these two drugs are safe together with minimal side effects.

Table 1. Risk of adenomas; evidence of substantial effect in active arm

	Follow-up colonoscopy 2 to 39 mo after beginning treatment (*n* = 267)		Follow-up colonoscopy 33 to 36 mo after beginning treatment (*n* = 204)	
	Placebo (*n* = 129)	DFMO/sulindac (*n* = 138)	Placebo (*n* = 97)	DFMO/sulindac (*n* = 107)
Detection of any adenoma				
Cumulative incidence of adenomas detected at end of the treatment (%)	53 (41.1)	17 (12.3)	42 (43.3)	12 (11.2)
Risk ratio* (95% CI)		0.30 (0.18-0.49)		0.26 (0.15-0.46)
P		<0.001		<0.001
Detection of advanced adenomas†				
Cumulative incidence of advance adenomas detected at end of the treatment (%)	11 (8.5)	1 (0.7)	9 (9.3)	1 (0.9)
Risk ratio* (95% CI)		0.085 (0.011-0.65)		0.10 (0.013-0.78)
P		0.001		0.004
Detection of advanced adenomas with size ≥1 cm				
Cumulative incidence of advanced adenomas with size ≥1 cm detected at end of the treatment (%)	9 (7.0)	1 (0.7)	7 (7.2)	1 (0.9)
Risk ratio* (95% CI)		0.10 (0.013-0.81)		0.13 (0.016-1.03)
P		0.004		0.02
Detection of multiple adenomas (>1)				
Patients with >1 adenoma, incidence (%)	17 (13.2)	1 (0.7)	15 (15.5)	1 (0.9)
Risk ratio* (95% CI)		0.055 (0.0074-0.41)		0.060 (0.0081-0.45)
P		<0.001		<0.001
Sensitivity analysis imputing adenoma for patients without an end-point determination‡				
Cumulative incidence of adenomas detected at end of the treatment (%)	76/184 (41.3)	39/191 (20.4)		
Risk ratio* (95% CI)		0.49 (0.36-0.69)		
P		<0.001		

*Relative risk estimation by log-binomial regression. Likelihood ratio test *P* values are reported.
†Advanced adenomas in the placebo group included tubulovillous (3), intramucosal carcinoma (2), size ≥1 cm (6), and one serrated adenoma with high-grade dysplasia; the one advanced adenoma in the treatment group was an adenoma >1 cm.
‡Sensitivity analysis imputing adenoma for all patients without an end-of-study colonoscopy at the placebo rate of recurrence.

Cancer Prev Res, 2008, 1:32-8

Specific experience with DFMO and studies in patients with adenomatous polyps are described in four clinical chemoprevention studies that have been performed (59, 60, 61, 62)

Boyle JO et al in a pilot study established that DFMO could lower polyamine content in colorectal mucosa. They demonstrated that shed oral extracellular mucosal cells did not provide a reliable surrogate estimate of DFMO effects on the colon. Meyskens et al

performed a dose de-escalation chemoprevention trial of 2 difluoromethylornithine in patients with colon polyps. The short-term (one month) Phase IIa study established that DFMO was both safe and effective in reducing colorectal mucosal polyamine contents when administered orally to patients as low as .25 gm/m^2 for 28 days. No ototoxicity was observed at doses up to twice this amount. They demonstrated that both putrescine content and the ratio of spermidine to spermine and changes in these parameters as a function of DFMO treatment decreased as a function of donor age, which is an important consideration in the evaluation of DFMO effect. Meyksens then showed in an intermediate term (12 months) Phase IIb clinical chemoprevention trial demonstrated that polyamine levels in rectal mucosa can be continually suppressed by daily oral doses of DFMO that produced few or no side effects (62). Using a similar design, a smaller study has confirmed these results (63); this trial reported some reversible ototoxicity (at a dose of DFMO of 500 mg/m^2/day).

Usage of DFMO at high doses for therapeutic purposes indicated that hearing loss occurred and seemed to be related to the total dose; this toxicity was however reversible (64). Therefore the toxicity of low dose DFMO has been assessed in a series of pilot, phase IIa and phase IIb trials and detailed the evaluation of hearing changes described in the phase IIb trial (65). They were unable to demonstrate an effect of DFMO on otoacoustic emissions at any dose of DFMO (0, -75, 200, 400 mg/m^2/day) administered. They detected a subtle decrease in pure tone threshold that was dose-related. However, even at the highest dose the decrease was less than 4 db. Since 1-2 db decreases per year of hearing in normal humans in this age range have now been shown (66,67), results should not raise undue concern since: (1) the effects of DFMO on hearing loss are known to be rapidly reversible after discontinuation of the drug; (2) 10-20 db losses need to occur before the effect is clinically evident. Nevertheless, all participants will receive a baseline audiogram, throughout the study and if clinically indicated.

Clinical experience with Etoposide.

Etoposide has been used extensively in pediatric neuroblastoma. It is used in upfront therapy in combination with cisplatin. It has been used in the relapse setting alone and in combination with many chemotherapeutic agents including combinations with topotecan, cytoxan, cisplatin, ifosphamide, vincristine. It is used safely as relapsed neuroblastoma therapy at 50 mg/m2/day for 10 to 21 days (72,73). These studies showed antineuroblastoma effects in patients with asymptomatic relapses but no effect in rapidly progressing neuroblastoma.

Summary

These studies suggest that ODC/polyamines are critical in oncogenesis and therefore present a therapeutic target for the treatment and prevention of recurrence of NB and other types of cancer. In addition to ODC, other targets in the polyamine pathway include AdoMetDC and also the polyamine uptake system.

This study will focus on the combination of DFMO with etoposide given increased effect of this combination in vitro and strong experience of etoposide in neuroblastoma. Patients

will have received etoposide in 2 early cycles of upfront therapy. This will not preclude patients from use in the relapsed setting as the dose used in this trial has been shown effective in relapsed neuroblastoma patients. Etoposide is not currently being used in a relapsed neuroblastoma clinical trial and therefore is felt to be an excellent candidate for this patient population.

3.0 Study Objectives

The primary objective is to evaluate the MTD of DFMO alone and with etoposide in these patients based on defined dose escalation schema.

3.1 Primary Objective:

3.1.1 Test the safety of DFMO in children with relapsed or refractory neuroblastoma alone and in combination with etoposide.

3.2 Secondary Objectives:

3.1.2 To evaluate progression free survival and response.
3.1.3 Evaluate the pharmacologic serum levels of DFMO during Cycle 1 and 2, days 1 & 8.
3.1.4 Biology studies to include: effect on polyamine depletion, ODC activity, genomic analysis of cells pre- and post- treatment, correlation of *in vitro* response to *in vivo* response, flow cytometry of tumor burden in bone marrow and biomarker development.

4.0 Study Design

This study is an open label, multicenter, dose escalation study in patients with refractory or recurrent neuroblastoma.

4.1 Cycle 1 : DFMO

Three patients will be enrolled to receive single agent DFMO PO administered on Days 1-21 of the first 21-day cycle. The starting dose is 500 mg/m^2 PO BID(Dose Level 1). Dose escalation will take place in a standard 3+3 design, in which doses will increase by approximately 20 to 25% in successive 3-patient cohorts.

Enrollment of the next cohort will occur after the entire previous cohort has completed both cycles 1 (single agent) and 2 (combination) of treatment without any dose limiting toxicity (DLT). If a DLT is observed in one out of three patients during these cycles, an additional three patients will be enrolled to receive the same dose of DFMO. If a DLT is observed in another patient (a total of two out of six patients), three additional patients will be enrolled to receive DFMO at the next lower dose level. The

MTD of single agent DFMO and combination will be defined as the dose level below which DLTs are seen in ≥2 of at least six patients dosed.

4.2 Combination Cycles (Cycles 2-5): DFMO with Etoposide.

After the first cycle of single agent DFMO, patients will receive etoposide in Cycle 2-5 in addition to DFMO. The dose of DFMO will be the safe and tolerated dose administered in the first cycle.

Drug doses and schedule for etoposide: Etoposide will be given at 50mg/m2/dose PO daily for the first 14 days of each 21 day cycle. Capsules will be rounded to closest 50 mg.

4.3 Response Evaluations

At the times indicated in Section 7.0 and the Table of Procedures and Assessments, scans will be obtained to evaluate response data for patients enrolled in this study. Response will be assessed according to criteria outlined in Section 9.0 (Data Analysis) to evaluate the activity and potential benefit of DFMO as well as etoposide in this patient population.

4.4 Pharmacokinetics

Plasma concentrations of DFMO will also be determined during cycle 1 and cycle 2 on Days 1 and 8 at hours 0(pre dose), 30min, 1hr, 3hr, 6 hrs.

4.5 Biological Studies

Subjects will have an opportunity to participate in correlative biological studies on a voluntary basis. This study will evaluate the level of polyamines and ODC activity in tumor cells pre and post treatment. This study may be able to contribute to our knowledge of molecular determinants of response to therapy and/or development of biomarkers to help guide future therapy.

5.0 Patient Selection

Eligibility:

5.1 ***Inclusion Criteria:***

5.1.1 Age: 0-21 years at the time of diagnosis.
5.1.2 Diagnosis: Histologic verification at either the time of original diagnosis or relapse of neuroblastoma.
5.1.3 Disease Status: Refractory or relapsed neuroblastoma
5.1.4 Measurable disease, including at least one of the following:
5.1.4.1 Measurable tumor >10mm by CT or MRI
5.1.4.2 A positive MIBG and abnormal urinary catecholamine levels
5.1.4.3 Positive bone marrow biopsy/aspirate.
5.1.5 Current disease state must be one for which there is currently no known curative therapy.
5.1.6 A negative urine pregnancy test is required for female subjects of child bearing potential (onset of menses or ≥13 years of age).
5.1.7 Patients without bone marrow metastases must have an ANC > 500/µl and platelet count >50,000/µl
5.1.8 Organ Function Requirements
5.1.7.1 Subjects must have adequate liver function as defined by AST and ALT <10x upper limit of normal
5.1.7.2 Serum bilirubin must be ≤ 2.0 mg/dl
5.1.7.3 Serum creatinine must be ≤ 3 x upper limit of normal
5.1.8 Informed Consent: All subjects and/or legal guardians must sign informed written consent. Assent, when appropriate, will be obtained according to institutional guidelines

5.2 ***Exclusion Criteria:***

5.2.1 Life expectancy <2 months or Lansky score <30%
5.2.2 Investigational Drugs: Subjects who are currently receiving another investigational drug are excluded from participation.
5.2.3 Anti-cancer Agents: Subjects who are currently receiving other anticancer agents are not eligible. Subjects must have fully recovered from the effects of prior chemotherapy (hematological and bone marrow suppression effects)
5.2.4 Infection: Subjects who have an uncontrolled infection are not eligible until the infection is judged to be well controlled in the opinion of the investigator.
5.2.5 Subjects who, in the opinion of the investigator, may not be able to comply with the safety monitoring requirements of the study, or in whom compliance is likely to be suboptimal, should be excluded.

Protocol Design Schema

*Initial Evaluation**

↓

DFMO at Dose Level
orally
every 21 days (Cycle 1)

DFMO dose escalation:
Level 1 = 500mg/m2 PO BID
Level 2 = 750mg/m2 PO BID
Level 3 =1000mg/m2 PO BID
Level 4 =1500mg/m2 PO BID

↓

Re-evaluation

↓

DFMO at Dose Level
With
Etoposide 50mg/m2 PO daily for first 14 days of
every 21 day cycle
(Cycles 2-5)

↓

Continue to repeat with reevaluations after each 2 cycles

***Evaluation Includes: 1. Response evaluation: CT, MIBG, VMA, Bone Marrow 2. Biological evaluation:** Tumor cells isolated from bone marrow will have evaluation of: MYCN status, Polyamine levels, ODC activity.

6.0 STUDY DRUG ADMINISTRATION

6.1 DFMO

6.1.1 Description and Formulation of DFMO

DFMO powder will be provided in 75 mL amber glass bottles with a tamper-proof seal and child-resistant cap. The fill sizes are 75mg, 300mg, 450mg, and 1000 mg. There are no excipients added.

6.1.2 Dose Calculation and Administration of DFMO

Treatment will be administered on an outpatient basis unless hospitalization is required for another reason. The dose escalation of DFMO will take place in successive cohorts as follows:

DFMO Dose Level	Dose
1	500 mg/m^2 PO BID
2	750 mg/m^2 PO BID
3	1000 mg/m^2 PO BID
4	1500 mg/m^2 PO BID

6.1.3 DFMO Dosing Table Per Cohort:

The following tables will be used to determine dispensing of DFMO. All dosing calculations are within 10% of dose level.

DFMO Dose Level 1 – 500mg/m2 PO BID:

BSA (m2)	Bottles to be Dispensed for each dose	Actual Mg/m2
0.7	One 75mg bottle + One 300mg bottle =375mg (per dose)	528
0.8	One 75mg bottle + One 300mg bottle =375mg (per dose)	469
0.9	One 450mg bottle =450mg (per dose)	500
1	One 75mg bottle + One 450mg bottle =525mg (per dose)	525
1.1	One 75mg bottle + One 450mg bottle =525mg (per dose)	477
1.2	Two 300mg bottles =600mg (per dose)	500
Other	Contact Principal Investigator for correct dosing	

DFMO Dose Level 2 – 750mg/m2 PO BID:

BSA (m2)	Bottles to be Dispensed for each dose	Actual Mg/m2
0.7	One 75mg bottle +<br>One 450mg bottle<br>= 525mg (per dose)	750
0.8	Two 300mg bottles<br>=600mg (per dose)	750
0.9	Two 300mg bottles +<br>One 75mg bottle<br>=675mg (per dose)	750
1	One 300mg bottles +<br>One 450mg bottles<br>=750mg (per dose)	750
1.1	Two 300mg bottles +<br>Three 75 bottles<br>=825mg (per dose)	750
1.2	Two 450mg bottles<br>= 900mg (per dose)	750
Other	Contact Principal Investigator for correct dosing	

DFMO Dose Level 3 – 1000mg/m2 PO BID:

BSA (m2)	Bottles to be Dispensed for each dose	Actual Mg/m2
0.7	Two 300mg bottles+<br>One 75mg bottle<br>=675mg (per dose)	964
0.8	One 450mg bottle +<br>One 300mg bottles +<br>One 75mg bottles<br>=825mg (per dose)	1031
0.9	Two 450mg bottles<br>= 900mg (per dose)	1000
1	One 1000mg bottle<br>= 1000mg (per dose)	1000
1.1	One 1000mg bottle+<br>One 75mg bottle<br>= 1075mg (per dose)	977
1.2	Four 300mg bottles<br>=1200mg (per dose)	1000
Other	Contact Principal Investigator for correct dosing	

DFMO Dose Level 4 – 1500mg/m2 PO BID:

BSA (m2)	Bottles to be Dispensed for each dose	Actual Mg/m2
0.7	Two 300mg bottles + One 450mg bottle =1050mg (per dose)	1500
0.8	Four 300mg bottles =1200mg (per dose)	1500
0.9	Three 450mg bottles =1350mg (per dose)	1500
1	One 1000mg bottle One 450mg bottle =1450mg (per dose)	1450
1.1	One 1000mg bottle + Two 300mg bottles =1600mg (per dose)	1600
1.2	Four 450mg bottles =1800mg (per dose)	1500
Other	Contact Principal Investigator for correct dosing	

6.1.4 Administration of DFMO

Each entire dose of DFMO powder dispensed should be mixed with 4oz-8oz of liquid until dissolved (more liquid may be used, but all liquid must be ingested. Too much liquid may be difficult to consume). The entire amount of liquid should be ingested.

Recommended liquids for mixing (to cover taste)- Lemonade, Apple, Cranberry, Grape, or Pineapple

DFMO may NOT be mixed in other citric juices such as orange or grapefruit juice.

DFMO may also be mixed in 1-2 tablespoons of chocolate syrup or peanut butter. The DFMO will not dissolve, and this is acceptable. This technique is simply to mask the flavor.

Subjects will be given a ‘patient instruction sheet’ for home administration.

Subjects will be given a ‘dosing diary’ to keep track of doses given at home.

6.1.5 Storage

DFMO should be stored at room temperature and away from light.

Drug Requirements and Dosing:
Dr. Meyskens has held an IND (#31,143) from the FDA to use DFMO in studies of its effects on preneoplasias for over 15 years. Phase IIa and IIb studies of DFMO for the prevention of colon polyps have already been completed and a phase IIb trial of DFMO and sulindac is nearly complete (under NCIs IND). A dose of 250 mg/M^2/day has produced clear evidence of polyamine suppression in colonic mucosa with minimal to no side effects being demonstrated (62).

Toxicity for DFMO Potential Risk:

Likely<br>Happens to 10-30 patients out of every 100	Less Likely<br>Happens to 3-10 patients out of every 100	Rare<br>Happens to fewer than 3 patients out of every 100
• Fewer red and white blood cells<br>o a low number of red blood cells can make you feel tired and weak and may require transfusion.<br>o a low number of white blood cells can make it easier to get infections<br>• Diarrhea<br>• Decrease in the number of platelets made in the bone marrow	• Skin Rash<br>• Hair Loss<br>• Nausea and Vomiting<br>• Hearing Loss	• Loss of appetite<br>• Abdominal Pain<br>• Dizziness<br>• Headache

Four particular areas of concern have been identified with regard to the safety of subjects participating in this study and an attempt to address each of them is outlined below: The main considerations are: thrombocytopenia, hearing loss, gastrointestinal and non-G.I. side effects.

a) DFMO Specific Adverse Events

(1) Hearing loss.

Although hearing loss has been a problem at higher doses (see below), clinical changes in hearing have been uncommon (one of 123 patients in the phase IIb trial) and reversible in the doses proposed for this trial (62). An extended analysis of these observations is in press (65). There was no statistically significant shifts in distortion product otoacoustic emission levels. For auditory pure tone thresholds, there was a subtle 2-3 dB decrease in hearing sensitivity for the two higher DFMO doses (0.2 and 0.4 gm/M^2/day), but only for the two lowest frequencies at 250 and 500 HZ. However in two phase I trials using lower doses of DFMO, done by other investigators, no audiometric changes were seen after approximately 6 months of DFMO therapy at 0.50Gm/m^2/d (total dose 90Gm) although some changes were seen at higher doses (79). Hearing loss may occur in association with

DFMO administration at high doses. In a meta-analysis of previous studies, (64), it was reported that less than 10% of the patients who received cumulative doses below 150 Gm/m^2 developed a demonstrable hearing deficit, while hearing losses were observed in up to 75% of patients who received cumulative doses above 250 Gm/m^2. This side effect was thought to be totally reversible upon drug discontinuation. Some study participants taking DFMO at doses similar to those used in this study have experienced mild decreases in hearing soft sounds. These changes have been uncommon and usually subclinical (that is, noticeable on special hearing tests called audiograms, but not in normal conversation or daily activities). Although an affected participant's level of hearing usually returns to its usual state when drug is stopped, in a small percentage of cases (occurring in less than 5% of participants taking the drug effects have persisted even after drug was stopped. These changes are most likely not reversible in all patients. However, in a recently completed phase IIb trial of DFMO they did not detect audiologic changes that were clinically significant, even in the highest dose group (0.4Gm/m^2/day) which represents a total dose of DFMO of 144 Gm/m^2 (extended analysis, 65). It is probable that at low doses of DFMO ongoing recovery of inner ear polyamines occurs and clinical hearing loss will be rarely, if ever, seen. Since hearing loss is usually totally reversible after drug discontinuation, this approach appears safe and cost effective.

(2) Thrombocytopenia (low platelets) Thrombocytopenia has been reported predominantly in studies using "therapeutic" doses of DFMO (>3Gm/m^2/day) and primarily in cancer patients who had previously undergone chemotherapy or patients with compromised bone marrow.. Other side effects ascribed to DFMO have been rare and, to date, seen only at high doses .

(3) Other: Skin rash, anemia, and neutropenia have also been seen with DFMO administration

6.2 Etoposide

6.2.1 Dose Calculation and Administration of Etoposide

Starting with Cycle 2, etoposide will be given at 50mg/m2/dose PO daily for the first 14 days of each 21 day cycle. Capsules will be rounded to closest 50 mg. For oral administration in children too young to take the capsules, the parenteral product can be used orally per section 6.2.2 below.

Subjects will be given a 'patient instruction sheet' for home administration including storage information.

Subjects will be given a 'dosing diary' to keep track of doses given at home

6.2.2 **ETOPOSIDE** (VP-16, VePesid, Etopophos) NSC #141540

Formulation and Stability: Available in 50mg pink capsules. Store capsules under refrigeration but do not freeze. Also available as a yellow solution with a pH of 3 to 4, available in100mg (5ml) or 500 mg (25 ml) multiple-dose sterile vials containing 20mg/per ml etoposide. Unopened vials of VP-16 are stable for 24 months at room temperature (25°C). Dilute with 0.9% sodium chloride injection or D5W. At room

temperature, the solution is thought to be stable for 48 hours at a concentration of 0.4mg/ml and for 96 hours at a concentration of 0.2mg/ml in both glass and plastic containers. At concentrations above 0.4mg/ml, the stability of the solution is highly unpredictable; therefore dilution to a concentration >0.4mg/ml is not recommended. DO NOT REFRIGERATE SOLUTION: keep agitation to a minimum. For oral administration in children too young to take the capsules, the parenteral product can be used orally. A 1:1 dilution (10 mg/mL) is stable for 3 weeks in Burron plastic oral syringes, and can be administered directly to be followed by sour candy or gum, or can be further diluted immediately prior to administration with fruit juice. Concentrations need to be 0.4 mg/mL or less to substantially enhance taste. At higher concentrations in fruit juice, precipitation may occur in less than 3 hours.

Source and Pharmacology: A semisynthetic derivative of podophyllotoxin that forms a complex with topoisomerase II and DNA which results in a single and double strand DNA breaks. Its main effect appears to be in the S and G2 phase of the cell cycle. The initial t1/2 is 1.5 hours and the mean terminal half-life is 4 to 11 hours. It is primarily excreted in the urine. There is poor diffusion into the CSF. The maximum plasma concentration and area under the concentration time curve (AUC) exhibit a high degree of patient variability. Etoposide is highly bound to plasma proteins (~94%), primarily serum albumin. Pharmacodynamic studies have shown that etoposide systemic exposure is related to toxicity. Preliminary data suggests that systemic exposure for unbound etoposide correlates better than total (bound and unbound) etoposide.

Etoposide Risks:

	**Common**<br>**Happens to 21-100 children out of every 100**	**Occasional**<br>**Happens to 5-20 children out of every 100**	**Rare**<br>**Happens to < 5 children out of every 100**
**Immediate:**<br>**Within 1-2 days of receiving drug**	**Nausea, vomiting**	**Decreased or loss of appetite, decreased blood pressure during the injection**	**Allergic reaction**
**Prompt:**<br>**Within 2-3 weeks, prior to next course**	**Decrease in the number of red and white blood cells and platelets made in the bone marrow**	**Hair loss (L), worsens side effects due to radiation treatments, diarrhea**	**Numbness, tingling, clumsiness, mouth sores, damage to the liver**
**Delayed:**<br>**Any time later during therapy, excluding the above conditions**			
**Late:**<br>**Any time after completion of treatment**			**A new cancer or leukemia resulting from this treatment**

6.3 Dose Limiting Toxicities and Guidelines for Dose Modifications

Dose limiting toxicities (DLT) for single agent DFMO will be made after Cycles 1 and 2 for determination of the maximum tolerated dose (MTD) of this treatment. Toxicities and dose modifications will be monitored in all cycles. Adjustments to the doses of study drug will be based upon toxicity, graded according to the NCI Common Toxicity Criteria (CTC), Version 3.0, if these were normal at baseline. Patients who have transfusion-dependent hematologic abnormalities due to bone marrow involvement pre-study will not be assessed by the hematologic criteria below and will not have DFMO doses held for hematologic reasons.. Events that are not described in the NCI criteria will be assigned grades according to the criteria provided in Section 10.0. Criteria for determining the relatedness of clinical adverse events to treatment (Section 10.0) should be utilized to determine the relationship of adverse events to the treatment.

6.3.1 Dose Modification (Cycles 1 and 2)

The events listed below constitute DLT criteria if they occur during cycles 1 or 2. Patients experiencing any toxicity specified below or any intolerable toxicity will have their dose of DFMO held until toxicities have reverted to ≤Grade 2 toxicity. Upon resolution of the toxicity, patients will receive a dose reduction of DFMO to previous cohort dose level:

- Grade 4 neutropenia or thrombocytopenia that persists for 7 days or longer <u>off study drug</u>
- Grade 3 elevation of transaminases that persists for 7 days or longer <u>off study drug</u>
- Any other Grade 3 non-hematologic toxicity, excluding alopecia or inadequately treated nausea, vomiting, diarrhea

If there is no resolution of toxicity by 14 days, DFMO should be discontinued, and patients should be discontinued from the study.

6.4 Guidelines for Individual Patient Dosing Modifications for Subsequent Cycles (Cycles 3 and beyond)

6.4.1 Dose Delays

<u>6.4.1.1 DFMO</u>

Patients experiencing any toxicity specified below or any intolerable toxicity will have their dose of DFMO held until toxicities have reverted to ≤Grade 2 toxicity. Upon resolution of the toxicity, patients will receive a dose reduction of DFMO to previous cohort dose level:

- Grade 4 neutropenia or thrombocytopenia (except for subjects that are bone marrow positive pre-study)
- Grade 3 elevation of transaminases

- Any other Grade 3 non-hematologic toxicity, excluding alopecia or inadequately treated nausea, vomiting, diarrhea

Treatment may be delayed no more than 14 days to allow recovery from toxicity; if after 14 days toxicity continues with no reduction in severity, the patient will be removed from the study.

6.4.1.2 Etoposide

Patients experiencing hematologic toxicity, related to etoposide, of ANC <750 will have etoposide held until their ANC is >750. If ANC<500 (grade 4), then DFMO will also be held per section 6.4.1.1.

6.4.2 Study Drug Accountability

Study drug must only be used for patients enrolled in the trial. The investigational site staff must maintain a careful inventory of study drug. Study drug use will be recorded on a Study Drug Inventory Form. This form will contain the following information:

- Patient number and initials for each patient receiving study drug
- Date and quantity of DFMO received by the site
- Date and quantity of DFMO dispensed
- Date and quantity of DFMO returned

At each monitoring visit, the clinical monitor will reconcile with the actual inventory of study drug at each site.

6.4.3 Concomitant Medications and Treatments

All intercurrent medical conditions will be treated at the discretion of the Investigator according to acceptable community standards of medical care. All concomitant medications and treatments will be documented on the appropriate case report form.
The following medications are not permitted during the trial:

- Any cytotoxic chemotherapy
- Any other investigational treatment
- Any other systemic anti-neoplastic therapy including, but not limited to, immunotherapy, hormonal therapy, targeted therapies, anti-angiogenic therapies, or monoclonal antibody therapy
- Any radiotherapy, including systemically administered radioisotopes, unless administered with palliative intent and not to an area that encompasses any target lesion being followed

Erythropoietin, blood products, anti-emetics, steroids, and transfusions may be administered at the discretion of the Investigator based on established criteria

7.0 STUDY PROCEDURES AND ASSESSMENTS

7.1 Enrollment of Patients

The Research Coordinator at the NMTRC (lead site) will keep a “possible enrollment list” for all sites combined. Patients will be allowed to enroll in the order that they are added to that list. Prior to consent of the patient, the research coordinator at the lead site will be contacted (via e-mail) to place patient on this possible enrollment list. This coordinator will then reply with study space availability. If a spot is not available at that time, the site will be contacted as soon as a spot does open up (based on the patients order on the list). If a spot is available at the time, the potential patient will undergo consent and completion of all required screening procedures and certification of all inclusion and exclusion criteria by the Investigator. If the patient fits all enrollment criteria, the site will again contact the coordinator at the lead site for official enrollment confirmation, dose level assignment, and unique patient identifier assignment. In addition, a study enrollment form will be faxed to the coordinator at the lead site. This determination will be made based on subject data (subject qualifies for study) and current subjects enrolled in trial (i.e. how many subjects are in the current cohort). A subject may NOT be enrolled on trial until official approval from the lead site is received and a dose assignment has been made. The lead site will contact all sites via e-mail at each point when a cohort has been met, so that the sites know enrollment is on hold until next cohort is approved to move forward. If a spot becomes available, the first patient from the list will be contacted and will have 2 weeks with which to enroll in the study. If this patient does not enroll by that time then the patient forfeits his/her spot and the next patient will be offered that spot.

All patients (or patients’ legal representatives) must provide written informed consent before any study specific assessments may be performed.

7.2 Screening

The Investigator is responsible for keeping a record of all patients screened for entry into the study and subsequently excluded.

The following screening procedures must be performed within 14 days (preferred) but not more than 21 days prior to the first dose of study drug and *after* last previous treatment for malignancy:

1. Signed informed consent form. All patients (or patients' legal representatives) must provide written informed consent before any study specific assessments may be performed. Signed informed consent form for voluntary participation in correlative biologic analysis.
2. CT or MRI of measurable disease sites
3. MIBG scan (not required if subject's disease is previously determined to be not MIBG avid)
4. Audiogram
5. Bone marrow aspirate and biopsy
6. For patients with additional informed consent, identification of archived tumor tissue (paraffin block or unstained slides) for use in correlative biologic studies

The following screening procedures must be performed up to 5 days prior to the first dose of study drug.

1. Complete medical and surgical history, including documentation of the histologic evidence of malignancy and prior treatments for cancer. Include all other pertinent medical conditions and a careful history of all prior medical treatments;
2. Demographics;
3. Physical examination (including height and weight), noting all abnormalities including baseline dermatologic and neurologic exam, sites of palpable neoplastic disease,
4. BSA calculation (from body weight and height);
5. Vital signs, including temperature, pulse rate, and blood pressure
6. ECOG Performance status/Lansky Play status (Appendix I);
7. CBC with differential;
8. Serum electrolytes, blood urea nitrogen (BUN), creatinine, bilirubin, LDH, ALT, AST and ferritin;
9. Urine catecholamines
10. Urine pregnancy test for female subjects of child bearing potential (onset of menses or ≥13 years of age);
11. Concomitant medications/therapies including documentation of steroid use and dose;
12. Confirmation of inclusion and exclusion requirements

Following completion of all required screening procedures and certification of all inclusion and exclusion criteria by the Investigator, the Lead site will be contacted (via e-mail or phone call), at which time the patient will be enrolled in the trial and a unique subject number assigned. The Lead Site will act as the central coordinating body, and as such, will then assign the subject to a specific dose level. The subject may not start on study until the Lead Site has provided official approval of enrollment and assigned the dose level.

7.3 Treatment Phase – Cycle 1

The first cycle will be 21 days in duration. The following procedures must be completed:

Cycle 1 Day 1 (may be performed up to 5 days prior to DFMO administration)

1. Physical examination (including body weight), focusing on an update of all previous abnormalities, any new abnormalities, and neurological exam;
2. Vital signs, including temperature, pulse rate, blood pressure (sitting);
3. Review and recording of concomitant medications;
4. Monitoring of AEs and review of concurrent illnesses
5. PK Samples Drawn per section 8.0
6. Urine catecholamines & polyamines
7. Dispense drug dosing diary

Cycle 1 Days 8 and 15 (+/- 3 day window)

Subjects will return to the clinic on Cycle 1 Day 8 and Cycle 1 Day 15 for evaluations. The following evaluations will be conducted:

1. Physical exam
2. Vital signs, including temperature, pulse rate, and blood pressure (sitting)
3. CBC with differential;
4. Serum electrolytes, BUN, creatinine, bilirubin, ALT, AST; and LDH
5. Review and recording of concomitant medications;
6. Monitoring of AEs and review of concurrent illnesses
7. Day 8 only- PK Samples drawn per section 8.0
8. Urine polyamines

End of Cycle 1

1. MIBG scan (for MIBG avid subjects only)
1. Bone marrow biopsy and aspirate (if positive at study entry) (If bone marrow is being done and patient has signed a voluntary informed consent for use of tissue in the correlative biologic study as a post-treatment sample please send additional samples per section 14.1.1)
2. CT/MRI (if used for radiologic assessment at baseline)
3. Audiogram

Any patient that has progression of disease (defined in section 9.0) at any point during Cycle 1 will not be required to go off study and may continue directly on to Cycle 2 (combination therapy) -all pre cycle 2 procedures must be completed prior to starting.

7.4 Treatment Phase – Cycle 2

The second cycle will be 21 days in duration. The following procedures must be completed:

Cycle 2 Day 1 (+/- 3 day window) (the following may be performed up to 5 days prior to cycle start)

1. Physical examination and neurological exam (including weight and height);
2. Vital signs, including temperature, pulse rate, blood pressure (sitting);
3. Review and recording of concomitant medications;
4. Monitoring of AEs and review of concurrent illnesses
5. BSA calculation (from body weight and height);
6. ECOG Performance status/Lansky Play status (Appendix I);
7. CBC with differential;
8. Serum electrolytes, blood urea nitrogen (BUN), creatinine, bilirubin, LDH, ALT, and AST;
9. PK Samples Drawn per section 8.0
10. Urine catecholamines and polyamines
11. Collect previous cycle drug dosing diary and dispense new drug dosing diary
12. Urine pregnancy test for female subjects of child bearing potential (onset of menses or ≥13 years of age);

Cycle 2 Days 8 and 15 (+/- 3 day window)

Subjects will return to the clinic on Cycle 2 Day 8 and Cycle 2 Day 15 for evaluations. The following evaluations will be conducted:

1. Physical exam- Required on Day 15 only
2. Vital signs, including temperature, pulse rate, and blood pressure (sitting)
3. CBC with differential;
4. Serum electrolytes, BUN, creatinine, bilirubin, ALT, AST; and LDH
5. Day 8 only- PK Samples drawn per section 8.0
6. Review and recording of concomitant medications;
7. Monitoring of AEs and review of concurrent illnesses

7.5 Treatment Phase – Cycle 3

The third cycle will be 21 days in duration. The following procedures must be completed:

Cycle 3 Day 1 (+/- 3 day window) (the following may be performed up to 5 days prior to cycle start)

1. Physical examination and neurological exam (including weight and height);;
2. Vital signs, including temperature, pulse rate, blood pressure (sitting);
3. Review and recording of concomitant medications;
4. Monitoring of AEs and review of concurrent illnesses
5. BSA calculation (from body weight and height);
6. ECOG Performance status/Lansky Play status (Appendix I);
7. CBC with differential;
8. Serum electrolytes, blood urea nitrogen (BUN), creatinine, bilirubin, LDH, ALT, and AST;
9. Urine catecholamines and polyamines
10. Collect previous cycle drug dosing diary and dispense new drug dosing diary
11. Urine pregnancy test for female subjects of child bearing potential (onset of menses or ≥13 years of age);

Cycle 3 Days 8 and 15 (+/- 3 day window)

Subjects will return to the clinic on Cycle 3 Day 8 and Cycle 3 Day 15 for evaluations. The following evaluations will be conducted:

1. Physical exam- Required on Day 15 only
2. Vital signs, including temperature, pulse rate, and blood pressure (sitting)
3. CBC with differential;
4. Serum electrolytes, BUN, creatinine, bilirubin, ALT, AST; and LDH
5. Review and recording of concomitant medications;
6. Monitoring of AEs and review of concurrent illnesses

End of Cycle 3

2. MIBG scan (for MIBG avid subjects only)
3. Bone marrow biopsy and aspirate (if positive at study entry) (If bone marrow is being done and patient has signed a voluntary informed consent for use of tissue in the correlative biologic study as a post-treatment sample please send additional samples per section 14.1.1)
4. CT/MRI (if used for radiologic assessment at baseline)
5. Audiogram

7.6 Treatment Phase – Cycle 4

The fourth cycle will be 21 days in duration. The following procedures must be completed:

Cycle 4 Day 1 (+/- 3 day window) (the following may be performed up to 5 days prior to cycle start)

1. Physical examination and neurological exam (including weight and height);;
2. Vital signs, including temperature, pulse rate, blood pressure (sitting);
3. Review and recording of concomitant medications;
4. Monitoring of AEs and review of concurrent illnesses
5. BSA calculation (from body weight and height);
6. ECOG Performance status/Lansky Play status (Appendix I);
7. CBC with differential;
8. Serum electrolytes, blood urea nitrogen (BUN), creatinine, bilirubin, LDH, ALT, and AST;
9. Urine catecholamines and polyamines
10. Collect previous cycle drug dosing diary and dispense new drug dosing diary
11. Urine pregnancy test for female subjects of child bearing potential (onset of menses or ≥13 years of age);

Cycle 4 Days 8 and 15 (+/- 3 day window)

Subjects will return to the clinic on Cycle 4 Day 8 and Cycle 4 Day 15 for evaluations. The following evaluations will be conducted:

1. Physical exam- Required on Day 15 only
2. Vital signs, including temperature, pulse rate, and blood pressure (sitting)
3. CBC with differential;
4. Serum electrolytes, BUN, creatinine, bilirubin, ALT, AST; and LDH
5. Review and recording of concomitant medications;
6. Monitoring of AEs and review of concurrent illnesses

7.7 Treatment Phase – Cycle 5

The fifth cycle will be 21 days in duration. The following procedures must be completed:

Cycle 5 Day 1 (+/- 3 day window) (the following may be performed up to 5 days prior to cycle start)

1. Physical examination and neurological exam (including weight and height);;
2. Vital signs, including temperature, pulse rate, blood pressure (sitting);
3. Review and recording of concomitant medications;
4. Monitoring of AEs and review of concurrent illnesses
5. BSA calculation (from body weight and height);
6. ECOG Performance status/Lansky Play status (Appendix I);
7. CBC with differential;
8. Serum electrolytes, blood urea nitrogen (BUN), creatinine, bilirubin, LDH, ALT, and AST;
9. Urine catecholamines and polyamines
10. Collect previous cycle drug dosing diary and dispense new drug dosing diary
11. Urine pregnancy test for female subjects of child bearing potential (onset of menses or ≥13 years of age);

Cycle 5 Days 8 and 15 (+/- 3 day window)

Subjects will return to the clinic on Cycle 5 Day 8 and Cycle 5 Day 15 for evaluations. The following evaluations will be conducted:

1. Physical exam- Required on Day 15 only
2. Vital signs, including temperature, pulse rate, and blood pressure (sitting)
3. CBC with differential;
4. Serum electrolytes, BUN, creatinine, bilirubin, ALT, AST; and LDH
5. Review and recording of concomitant medications;
6. Monitoring of AEs and review of concurrent illnesses

End of Cycle 5

1. MIBG scan (for MIBG avid subjects only)
6. Bone marrow biopsy and aspirate (if positive at study) (If bone marrow is being done and patient has signed a voluntary informed consent for use of tissue in the correlative biologic study as a post-treatment sample please send additional samples per section 14.1.1)
2. CT/MRI (if used for radiologic assessment at baseline)
3. Audiogram

7.8 Subsequent Cycles

Drug administration will be according to guidelines with premedication as specified. The following evaluations will be performed on the days of each cycle as indicated.

Day 1 of Each Cycle (up to 5 days pre cycle):

1. Physical exam and neurological exam
2. Monitoring of AEs and review of concurrent illnesses (treatment-emergent or worsening illness must be recorded as an adverse event);
3. Vital signs, including temperature, pulse rate, and blood pressure (sitting)
4. CBC with differential;
5. Serum electrolytes, BUN, creatinine, bilirubin, LDH, ALT, AST
6. Urine catecholamines
7. Review and recording of concomitant medications

7.9 Other Schedules:

1. Additional imaging or assessments may be done if clinically indicated by symptoms or exam. Type of assessment and timing should be recorded as well as reason for assessment.
2. Survival will be monitored on an ongoing basis during the study, and then every 3 months from the time the patient is off-study up to a period of 1 year from the patient's date of enrollment.
3. PK samples will be obtained at any time there is a toxicity judged related to study drug requiring dose modification (delay and/or reduction) as closely as possible to the event

7.10 Study Completion

Subjects who receive 5 total 21-day treatment cycles will be considered as having completed the study. Additional treatment cycles may be delivered in a maintenance setting if there are no safety concerns, there is no disease progression, and/or there is an indication of clinical benefit, after discussion with the Principal Investigator. Maintenance monitoring will be conducted as described for Subsequent Cycles, with the appropriate recording on CRFs.

7.11 Follow-up Visit

Subjects will return to the clinic 30 (+7) days after the last dose of DFMO, and the following evaluations will be conducted:

1. Physical examination (including body weight), focusing on an update of all previous abnormalities, any new abnormalities and neurological exam;
2. Vital signs, including temperature, pulse rate, blood pressure (sitting);
3. CBC with differential;
4. Serum electrolytes, BUN, creatinine, bilirubin, LDH, ALT, AST;
5. Urine catecholamines
6. Review and recording of concomitant medications;
7. Monitoring of AEs and review of concurrent illnesses
8. MRI/CT; MIBG scan; bone marrow biopsy and aspirate (if positive at study entry)
9. Audiogram (if clinically indicated)

Any subject with a suspected study drug-related toxicity at the follow-up visit must be followed until resolution or stabilization of the event. This may require additional clinical assessments and laboratory tests. The follow-up results will be recorded on the appropriate page of the CRF, as well as in the subject's source documentation.

7.12 Survival Follow-up

Subjects will be followed for long term survival by contact with parent or treating institution to confirm survival at the following time points (time from last dose of study drug):

- 3 months
- 6 months
- 9 months
- 1 year

The follow-up results will be recorded on the appropriate page of the CRF, as well as in the subject's source documentation.

8.0 PHARMACOKINETIC AND URINE STUDIES

8.1 Pharmacokinetic (PK) Sample Days:

On Days 1 and 8 of cycle 1 and 2- PK specimens will be collected.

- 8.1.1 The subject will hold (not take) their 1st dose of DFMO on days one and eight until arriving to the clinic and pre-dose blood specimen is collected.
- 8.1.2 Pre-dosing PK sample will be collected and processed as per Section 8.2 and 8.3
- 8.1.3 Once the pre-drug specimen is collected the subject will take their prescribed morning dose of DFMO.
- 8.1.4 +/- 5 min: 30 min. +/- 15 minutes: 1hr, 3 hr, and 6 hrs the subject's morning dose of DFMO, PK samples will be collected.
- 8.1.5 The date and time of all specimen collections and of the dose of DFMO will be noted in the subject's chart.

8.2 PK Sample requirements:

One 2cc whole blood specimen will be collected in a serum separator tube (SST).

8.3 Specimen Handling:

- 8.3.1 Following collection (as described in 8.2) the specimen will to be allowed to clot at room temperature for no less than twenty to thirty minutes.
- 8.3.2 Specimen will be spun in a centrifuge at 1,000- 1,300g for 10-15 minutes
- 8.3.3 Following centrifugation the serum from the SST will be aliquoted into a plastic screw capped cryovial.
- 8.3.4 Specimens must be labeled with the subject's initials, unique study identifier, and date and time of specimen collection.
- 8.3.5 Specimen will then be stored in a -70° C. freezer until ready to be shipped. Specimens will be batch shipped once site is approved to ship by the NMTRC.
- 8.3.6 Samples will be sent on dry ice to:

Ping Zhao
Neuroblastoma Translational Research Laboratory
Van Andel Research Institute -5th floor
333 Bostwick Ave
Grand Rapids, MI 49503
Ph: 616-234-5394
ping.zhao@vai.org

8.4 Urine Polyamines Collection:
On Days 1, 8, and 15 of Cycle 1 and on Day 1 of cycles 2-5 urine polyamines will be collected.

8.4.1 First morning void urines will be collected in containers and placed at 4°C until transported to the laboratory.
8.4.2 Specimens will be stored at -20°C until analysis for the dc-SAM and polyamines.
8.4.3 Samples will be batch shipped on a Monday (send after 20 samples are collected) and sent on dry ice to:
Arizona Cancer Center
1515 N. Campbell Ave.,
Tucson, AZ 85724
Phone: 520-626-3138
egerner@canprevent.com

9.0 Response Criteria

Overall response rate (ORR) in patients with radiologically assessable disease will be determined by CT or MRI by cross-sectional imaging, in neuroblastoma by MIBG scans, and/or bone marrow assessment.

Response Assessment: Each subject will be classified according to their "best response" for the purposes of analysis of treatment effect. Best response is determined from the sequence of the objective statuses described below.

Response Criteria for Patients with Solid Tumors: This study will use the following Response Evaluation Criteria in Solid Tumors.

- Measurable disease: The presence of at least one lesion that can be accurately measured in at least one dimension with the longest diameter at least 10 mm
- Measurable Disease Response will be assessed at end of Cycle 1(at the end of single agent therapy) and Cycle 2 (after the first cycle of combination therapy) and then at least every other cycle
- Serial measurements of lesions are to be done with CT or MRI, using the same method of assessment is to be used to characterize each identified and reported lesion at baseline and during follow-up.
- Quantification of Disease Burden The sum of the longest diameter (LD) for all target lesions will be calculated and reported as the disease measurement. For this evaluation, all scans will undergo central review at the University of Vermont.
- **Complete Response (CR):** Disappearance of all target lesions.
- **Partial Response (PR):** At least a 30% decrease in the disease measurement, taking as reference the disease measurement done to confirm measurable disease at study entry..
- **Stable Disease (SD):** Neither sufficient decrease to qualify for PR or sufficient increase to qualify for PD from study entry.

- **Progressive Disease (PD):** At least a 20% increase in the sum of the longest diameters of all target lesions compared to study entry, the appearance of unequivocal new lesions, or laboratory evidence of clinical progression (e.g., spread to bone marrow or increasing catecholamines).

Response Criteria for Patients with Bone Marrow Disease:

- Those patients with morphologic evidence of neuroblastoma by routine H and E staining (NSE staining only is not evaluable) will be evaluable to assess bone marrow response.
- **Complete response:** No tumor cells detectable by routine morphology on two subsequent bilateral bone marrow aspirates and biopsies done at least three weeks apart after study entry.
- **Progressive disease**: Tumor seen on morphology on two consecutive bone marrows done at least three weeks apart in patients who had NO tumor in bone marrow at study entry. (Note: Patient may be declared as progressive disease in bone marrow after only one diagnostic bone marrow at the discretion of the treating physician after discussion with the study chair.)
- **Stable disease**: Persistence of an amount of tumor in the bone marrow by morphology that does not meet criteria for either complete response or progressive disease.

Response Criteria for Patients with MIBG Positive Lesions

- Patients who have a positive MIBG scan at the start of therapy will be evaluable for MIBG response. The use of 123I for MIBG imaging is recommended for all scans. If this radioisotope is unavailable at the treating institution, the use of the same radioisotope for all MIBG scans for an individual patient is strongly encouraged. All MIBG's will be performed at the research institution and centrally reviewed at the University of Vermont.
- **Complete response** = complete resolution of all MIBG positive lesions
- **Partial response** = resolution of at least one MIBG positive lesion, with persistence of other MIBG positive lesions.
- **Stable disease** = no change in MIBG scan in number of positive lesions (includes patients who have same number of positive lesions but decreased intensity)
- **Progressive disease** = Development of new MIBG positive lesions
- The response of MIBG lesions will be assessed on central review using the Curie scale as outlined below. Central review responses will be used to assess response for study endpoint. (**NOTE**: This scoring is NOT required to be done by the research institution for end of course response assessments).
- The body is divided into 9 anatomic sectors for osteomedullary lesions, with a 10th general sector allocated for any extra-osseous lesions**.** In each region, the lesions are scored as follows. The **absolute extension score** is graded as: 0 = no site per segment, 1 = 1 site per segment, 2 = more than one site per segment, 3 = massive involvement (>50% of the segment). The absolute score is obtained by adding the score of all the segments. (See diagram of sectors below)

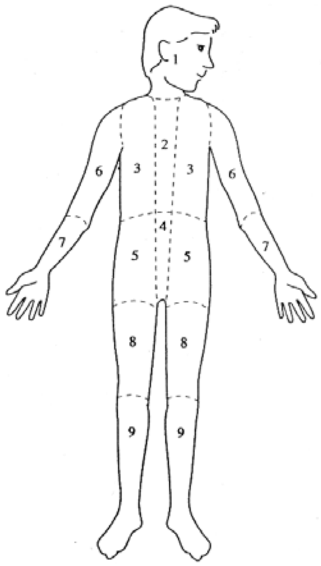

The **relative score** is calculated by dividing the absolute score at each time by the corresponding pretreatment overall score. The relative score of each patient is calculated at each response assessment and classified as below:

- **Complete response:** all areas of uptake on MIBG scan completely resolved.
- **Partial response**: Relative score < 0.2 (lesions almost disappeared) to < 0.5 (lesions strongly reduced).
- **Stable disease**: Relative score > 0.5 (lesions weakly but significantly reduced) to > 0.9 (lesions not reduced).
- **Progressive Disease**: New Lesions on MIBG scan.

Definition of Overall Response for Each Patient

This will be utilized as a basis to integrate response at all sites defined as measurable in this study, including CT/MRI lesions which meet criteria, MIBG positive lesions, and bone marrow disease. These criteria will be used to define the overall response for the patient in the statistical analysis.

- **Complete Response (CR)**
  Disappearance of all target lesions. No evidence of tumor at any site (chest, abdomen, liver, bone, bone marrow, nodes, etc.), and HVA/VMA normal.

- **Partial Response (PR)**
  At least a 30% decrease in the disease measurement for CT/MRI target lesions, taking as reference the disease measurement done to confirm measurable disease in target lesions at study entry. Bone marrow with CR. MIBG with either PR/CR in bone lesions; MIBG may be SD or CR in soft tissue lesions corresponding to lesions on CT/MRI. HVA/VMA may still be elevated.

- **Stable disease (SD)**
  The patient will be classified as stable disease if there are no new lesions; no new sites of disease, and they do not fit the criteria for PD/PR/CR as above.

- **Progressive Disease (PD)**
  Any one of the following:
  a) At least a 20% increase in the disease measurement for CT/MRI target lesions, taking as reference the smallest disease measurement recorded since the start of treatment.
  b) Appearance of one or more new lesions or new sites of tumor.
  c) PD as defined above for either bone marrow or MIBG lesions.

Duration of response:

Duration of response is defined as the period of time from when measurement criteria are met for complete response (CR) or partial response (PR), whichever is first recorded, until the first date that recurrent or progressive disease (PD) is objectively documented (taking as reference for PD the smallest measurements recorded since the treatment started)

The assessment of response will include the initial measurable targets and will be performed after the first and second cycle, then after every other cycle. Serial results of bone marrow aspirates, biopsies and urinary catecholamines will be reviewed for responding patients to confirm response or lack of progression.

The time to progression, defined as the period from the first day of administration of study drug until the criteria for progression are met taking as reference the screening measurements, will be assessed as Progression Free Survival (PFS).

Scan Submission:

All required study scans (CT's, MRI's, and MIBG's) will be reviewed by central radiology at the University of Vermont and Fletcher Allen Health Care according to section 9.0.

All study required scans should be sent on disc to:

Genevieve Bergendahl, RN
NMTRC
Van Andel Research Institute
5th Floor
333 Bostwick NE
Grand Rapids, MI 49503
Tel: (616) 234-5799
E-Mail: Genevieve.bergendahl@vai.org

10.0 Adverse Event Reporting

10.1 Definitions

10.1.1 Adverse Event

An ***adverse event*** is any untoward medical occurrence in a patient or clinical investigation subject administered a pharmaceutical product and which does not necessarily have to have a causal relationship with this treatment. An adverse event can therefore be any unfavorable and unintended sign (including an abnormal laboratory finding, for example), symptom, or disease temporally associated with the use of a medicinal product, whether or not considered related to the medicinal product.

An untoward medical event which occurs outside the period of follow-up as defined in the protocol will not be considered an adverse event unless related to study drug. Worsening of a medical condition for which the efficacy of the study drug is being evaluated will not be considered an adverse event.

10.1.2 Unexpected Adverse Event

An ***unexpected adverse event*** is one for which the nature or severity of the event is not consistent with the applicable product information, e.g., the investigator's brochure.

10.1.3 Serious Adverse Event

A ***serious adverse event*** is any untoward medical occurrence that at any dose:

- Results in death
- Is life-threatening (an event in which the patient was at risk of death at the time of the event; it does not refer to an event which hypothetically might have caused death if it were more severe)
- Requires in-patient hospitalization or prolongation of existing hospitalization
- Results in persistent or significant disability/incapacity
- Is a congenital anomaly or birth defect
- Other important medical events that may not be immediately life-threatening or result in death or hospitalization but may jeopardize the patient or may require intervention to prevent one of the other outcomes listed above. Examples of such events are intensive treatment in an emergency room for allergic bronchospasm; blood dyscrasias or convulsions that do not result in hospitalization; or development of drug dependency or drug abuse.

The term “severe” is often used to describe the intensity (severity) of an event; the event itself may be of relatively minor medical significance (such as a severe headache). This is not the same as “serious”, which is based on patient/event outcome or action criteria usually associated with events that pose a threat to a patient’s life or functioning.

10.1.4 Documenting Adverse Events

The Investigator should elicit information regarding the occurrence of adverse events through open-ended questioning of the patient, physical examination and review of laboratory results.

All Grade 2 or higher (per CTCAE version 3.0) adverse events, whether serious or not, will be described in the source documents and the adverse event page of the case report form. All new events (Grade 2 or higher), as well as those that worsen in intensity or frequency relative to baseline, which occur after administration of study drug through the period of protocol-specified follow-up, must be captured.

Information to be reported in the description of each adverse event includes:

- A medical diagnosis of the event (if a medical diagnosis cannot be determined, a description of each sign or symptom characterizing the event should be recorded)
- The date of onset of the event
- The date of resolution of the event and whether the event is serious or not
- Action taken; drug treatment required; non-drug treatment required; hospitalization or prolongation of hospitalization required; diagnostic procedure performed; patient discontinued from the study
- Outcome: complete recovery or return to baseline; unknown/lost to follow-up; adverse event persisting; patient died (notify lead site immediately, and complete the Serious Adverse Event page and the Final Visit section of the case report form)

Adverse events, regardless of suspected cause, will be collected for 30 days following the last dose of DFMO.

All adverse events will be reviewed by the safety officer, and adverse event CRF forms will be signed off by safety officer after the 30 days post off study date of each subject.

10.1.5 Expedited Reporting of Adverse Events

All fatal or life–threatening adverse events must be reported to the lead site immediately by telephone, fax, or e-mail within 24 hours of discovery as well as to the appropriate regulatory authorities (local IRB and FDA if required). The lead site will then report directly to the safety officer. If full information is not known, additional follow-up by the Investigator will be required.

All other serious adverse events must be reported to the lead site by telephone, fax, or e-mail within 5 days as well as to the appropriate regulatory authorities (local IRB and FDA if required).

The Investigator must report all serious adverse events reported to regulatory authorities in an expedited manner to the local IRB or IEC. All serious adverse events must be followed until resolution or stabilization.

10.2 Grading and Relatedness of Adverse Events

10.2.1 Grading of Severity of an Adverse Event

Each adverse event (Grade 2 or higher) will be graded for severity per the National Cancer Institute Common Terminology Criteria for Adverse Events (CTCAE V 3.0), and these criteria must be used in grading the severity of adverse events. The criteria can be found at: http://ctep.cancer.gov/reporting/ctc.html.

Grading of Severity of an Adverse Event Not Listed in Published Criteria:
For those adverse events which are not listed as part of the NCI CTCAE V 3.0, the same grading system should be used, where:

- **Mild** corresponds to an event not resulting in disability or incapacity and which resolves without intervention
- **Moderate** corresponds to an event not resulting in disability or incapacity but which requires intervention
- **Severe** corresponds to an event resulting in temporary disability or incapacity and which requires intervention
- **Life-threatening** corresponds to an event in which the patient was at risk of death at the time of the event
- **Fatal** corresponds to an event that results in the death of the patient

10.2.2 Relatedness to Study Drug

The Investigator must attempt to determine if an adverse event is in some way related to the use of the study drug. This relationship should be described as follows:

- ***Unlikely***: The event is clearly due to causes distinct from the use of the study drug, such as a documented pre-existing condition, the effect of a concomitant medication, or a new condition which, based on the pathophysiology of the condition, and the pharmacology of the study drug, would be unlikely related to the use of the study drug
- ***Possible:*** The event follows a reasonable temporal sequence from administration of the study drug and the event follows a known response pattern to the study drug BUT the event could have been produced by an intercurrent medical condition which, based on the pathophysiology of the condition, and the pharmacology of the study drug, would be unlikely related to the use of the study drug OR the event could be the effect of a concomitant medication

- ***Probable:*** The event follows a reasonable temporal sequence from administration of the study drug, the event follows a known response pattern to the study drug AND the event cannot have been reasonably explained by an intercurrent medical condition OR the event cannot be the effect of a concomitant medication
- ***Definite:*** The event follows a reasonable temporal sequence from administration of the study drug, the event follows a known response pattern to the study drug and based on the known pharmacology of the study drug, the event is clearly related to the effect of the study drug. The adverse event improves upon discontinuation of the study drug and reappears upon repeat exposure.
- ***Unknown:*** Based on the evidence available, causality cannot be ascribed

11.0 Patient Withdrawal and Trial Discontinuation

11.1 Criteria for Patient Withdrawal

Patients may be withdrawn from the study for the following reasons:

- Progressive neoplastic disease after completing cycle 2
- Patient or guardian withdraws consent to continue in the trial
- Patient develops an intercurrent illness that precludes further participation, or requires a prohibited concomitant treatment
- The Investigator withdraws the patient in the patient's best interests
- Patient is lost to follow-up (defined as the inability to contact the patient on 3 separate occasions over a period of 2 weeks)
- Administrative reasons (e.g., the patient is transferred to hospice care)
- An adverse event, which in the opinion of the Investigator, precludes further trial participation or fulfills the protocol requirements for withdrawal (e.g., the development of dose limiting toxicity despite a reduction in protocol therapy for a previous episode of dose limiting toxicity)
- Death

11.2 Patient Replacement

Patients who discontinue the trial before completing the second cycle of treatment for any reason other than DLT will be replaced to complete the number of evaluable patients required for determination of safety. All patients who receive one dose of DFMO will be included in the safety analysis.

11.3 Trial Discontinuation

The lead site may discontinue the trial as a whole or at an individual investigational site at any time. Reasons for early trial discontinuation may include, but are not limited to, unacceptable toxicity of study drug, a request to discontinue the trial from a regulatory authority, protocol violations at an investigational site, violations of good clinical practice at an investigational site, or poor enrollment. The lead site will promptly inform all Investigators in the event of premature study discontinuation and provide all Investigators with instructions regarding the disposition of patients still on study. Should the study be

terminated prematurely, all unused study drug, case report forms and any other study material will be returned to the lead site.

12.0 DATA ANALYSIS

12.1 Data Quality Assurance

Case report forms will be checked for correctness against source document data by the monitor. If any entries into the CRF are incorrect, incomplete or illegible, the monitor will ask the Investigator or the study site staff to make appropriate corrections. Once the CRF page is complete, it will be delivered to the lead site. Prior to this, the completed CRF will again be reviewed for completeness, consistency and legibility.

12.2 Statistical Analysis

This is an open label, Phase 1, exploratory trial evaluating the safety of DFMO as a single agent in a predominantly pediatric population, the safety of combination of DFMO with etoposide in this population, and evaluation of anti-tumor effects.

The following data sets will be used in this study:

• All enrolled and eligible subjects (ITT) population: All eligible patients who have a signed informed consent form.

•All treated and eligible patients (Safety and Response evaluable) population: All patients who received at least one dose of study drug

• All eligible patients treated ≥ 1 Cycle (as Treatment) population: All patients who received at least one cycle of study drug

• All eligible patients treated ≥ 2 Cycles (as Combination Treatment) population: All patients who received at least one cycle of DFMO and one cycle of DFMO and etoposide.

Response analyses will be performed on the Safety and Response evaluable population and sensitivity analyses will be performed on the ITT and as Treated populations to assess the impact of replacing non-evaluable patients and of including patients receiving less than 1 cycle. Safety analysis will be performed on the Safety and Response evaluable population.

Patients who are alive without documented evidence of disease progression will be determined to be “progression free” at each study time point. As well, progression-free survival will be calculated from the date of Day 1 Cycle 1 to the date that criteria for progression of disease are first seen.

The objective response rate, which will include all patients who experience CR or PR will be analyzed for all patients completing at least one cycle of treatment with DFMO and defined by the independent reading center.

PFS will be estimated using the Kaplan-Meier method with the median time to progression and its 95% confidence summarized.

13.0 ADMINISTRATIVE PROCEDURES

13.1 Patient Informed Consent

No study related procedures will be performed until a patient or a patient's legal representative has given written informed consent. The lead site will provide to the site Investigators a sample informed consent document that conforms to all the requirements for informed consent according to ICH GCP and US FDA guidelines (21 CFR 50). However, it is up to each site Investigator to provide a final informed consent that may include additional elements required by the Investigator's institution or local regulatory authorities. The IRB/EC for each investigational site must approve the consent form document prior to study activation; changes to the consent form during the course of the study may also require IRB/EC approval. The informed consent document must clearly describe the potential risks and benefits of the trial, and each prospective participant must be given adequate time to discuss the trial with the Investigator or site staff and to decide whether or not to participate. Each patient who agrees to participate in the trial and who signs the informed consent will be given a copy of the signed dated and witnessed document. The original copy of the signed, dated, and witnessed informed consent document will be retained by the Investigator in the study files.

The Investigator must also obtain authorization from the patient to use and/or disclose protected health information in compliance with the Health Insurance Portabiltiy and Accountability Act (HIPAA). Written HIPAA authorization may be obtained as part of the informed consent process.

13.2 Ethical Conduct of the Study and IRB/IEC Approval

The study will be conducted according to the principles of the 2004 version of the Declaration of Helsinki, the International Conference on Harmonization Guidance on Good Clinical Practice and the requirements of all local regulatory authorities regarding the conduct of clinical trials and the protection of human subjects.

The Investigator will submit the protocol, the Investigator's Brochure, the informed consent and any other material used to inform patients about the trial to the local IRB/IEC for approval prior to enrolling any patient into the trial. The IRB/IEC should be duly constituted according to applicable regulatory requirements. Approval must be in the form of a letter signed by the Chairperson of the IRB/IEC or the Chairperson's designee, must be on IRB/IEC stationary and must include the protocol by name and/or designated number. If an Investigator is a member of the IRB/IEC, the approval letter must stipulate that the Investigator did not participate in the final vote, although the Investigator may participate in the discussion of the trial. The Investigator will also inform the IRB/IEC of any serious adverse events that are reported to regulatory authorities and will provide to the IRB/IEC a final summary of the results of the trial at the conclusion of the trial.

Any amendments to the protocol will be done at the lead site, and will be submitted to the IRB/IEC for review and written approval before implementation. These approved changes will then be forwarded to sites for review by their local IRB/IEC. Written approval from sites will be forwarded to the lead site.

13.3 Data Safety Monitoring Board (DSMB)

An independent Data Safety and Monitoring Board (DSMB) will oversee the conduct of the study. The members of this Board will receive database summaries, including adverse event reports, and will convene either in person or via teleconference upon reaching enrollment of each cohort or every 4 months, whichever occurs first. The Board will be responsible for decisions regarding possible termination and/or early reporting of the study.

13.4 Monitoring

A clinical monitor will make regularly scheduled trips to the investigational site to review the progress of the trial. The actual frequency of monitoring trips will depend on the enrollment rate and performance at each site. At each visit, the monitor will review various aspects of the trial including, but not limited to, screening and enrollment logs; compliance with the protocol and with the principles of Good Clinical Practice; completion of case report forms; source data verification; study drug accountability and storage; facilities and staff.

During scheduled monitoring visits, the Investigator and the investigational site staff must be available to meet with the study monitor in order to discuss the progress of the trial, make necessary corrections to case report form entries, respond to data clarification requests and respond to any other trial-related inquiries of the monitor.

13.5 Pre-Study Documentation

Prior to initiating the trial, the Investigators at each site will provide to the Lead site the following documents:

- A signed FDA Form 1572
- A current (within 2 years), dated and signed curriculum vitae for the Principal Investigator and each sub-investigator listed on the FDA Form 1572
- A copy of the Investigator’s medical license from the state in which the study is being conducted; the Investigator’s medical license number on the Investigator’s c.v. will suffice as evidence of a license to practice medicine
- A letter from the IRB or EC stipulating approval of the protocol, the informed consent document and any other material provided to potential trial participants with information about the trial (e.g., advertisements)
- A copy of the IRB- or EC-approved informed consent document

- Current IRB membership list for IRB's without a multiple project assurance number or an IRB organization number under the Federal Wide Assurance program (www.ohrp.osophs.dhhs.gov).
- A signed Investigator Protocol Agreement
- A completed financial disclosure form
- Current laboratory certification for the reference laboratory
- A list of current laboratory normal values for the reference laboratory

13.6 Confidentiality

It is the responsibility of the investigator to insure that the confidentiality of all patients participating in the trial and all of their medical information is maintained. Case report forms and other documents submitted must never contain the name of a trial participant. Each patient in the trial will be identified by a unique identifier that will be used on all CRF's and any other material submitted to the lead site. All case report forms and any identifying information must be kept in a secure location with access limited to the study staff directly participating in the trial.

The results of the study may be presented in reports, published in scientific journals or presented at medical meetings; however, patient names will never be used in any reports about the study.

13.7 Source Documents

The Investigator will maintain records separate from the case report forms in the form of clinical charts, medical records, original laboratory, radiology and pathology reports, pharmacy records, etc. The Investigator will document in the clinic chart or medical record the name and number of the trial and the date on which the patient signed informed consent prior to the patient's participation in the trial. Source documents must completely reflect the nature and extent of the patient's medical care, and must be available for source document verification against entries in the case report forms when the monitor visits the investigational site. All information obtained from source documents will be kept in strict confidentiality.

13.8 Record Retention

The Investigator will retain the records of the study for 15 years, or for 2 years following the date that a marketing application for the study drug is approved, or if no marketing application is filed, or if such an application is not approved, for 2 years after the IND has been closed. The lead site will notify Investigators when retention of study records is no longer required. All study records must be maintained in a safe and secure location that allows for timely retrieval, if needed.

Study records that must be retained include copies of case report forms, signed informed consents, correspondence with the IRB or IEC, study drug dispensing and inventory

records, source documents, clinic charts, medical records, laboratory results, radiographic reports) and screening/enrollment logs.

Should the Investigator relocate or retire, or should there be any changes in the archival arrangements for the study records, the lead site must be notified. The responsibility for maintaining the study records may be transferred to another suitable individual, but the lead site must be notified of the identity of the individual assuming responsibility for maintaining the study records and the location of their storage.

14.0 Biological Evaluation

A major challenge in the development of cancer therapeutics is an absence of understanding the relationship of the disease to response to therapy, and the ability to predict which patients are most likely to respond to any particular agent. Emerging technologies including ability to establish primary tumor cells in culture, evaluate pharmacogenomics and explore biomarkers may provide a way to explore relationships between clinical benefit and treatment. As an optional component to this trial, patients have the opportunity to voluntarily participate in this parallel study aimed at developing methods to help understand clinical outcomes in neuroblastoma. Bone marrow samples will be collected per section 8.5

Isolation of Tumor Cells from Bone Marrow Aspirates. To isolate tumor cells from bone marrow aspirates, samples will be kept cold while dissociating. Two ml of bone marrow sample will be applied to a 40 µM strainer and the sample rinsed with ice cold HBSS until there is no more blood on filter (typically 50-300 ml). Then the filter is moved to new tubes as needed. Filter will be inverted over a clean 50 ml tube and neurospheres rinsed into the tube with ice cold HBSS. The sample will be divided into 15 ml tubes and spun at 1,000 RPM for 20 minutes. The supernatant will be aspirated and 2 ml of fresh media added to the sample, followed by mechanical trituration with a 10 ml pipette lined with FBS to prevent cells from sticking to the pipette. After samples settle, the supernatant is removed into new collection tubes. Two ml of fresh media will be added to settled tubes followed by re-trituration as necessary to break up large neurospheres, clots, and tissue. Tubes will be filled to 15 ml with media and mixed well by inversion, followed by centrifugation at 1,200 RPM for 10 minutes. After removal of all but 3 ml of supernatant, the remaining sample is triturated with a fire-narrowed pasteur pipette. Cells will be strained through 70 µM nylon mesh into a fresh 50 ml tube, washing tube and mesh several times with media and transferred to15 ml tubes, washing the cells from the 50 ml tube. Tubes will be filled with media and centrifuged at 1,200 RPM for 10 minutes. The supernatant will be aspirated, leaving 1-2 ml media which is suspended in neurobasal medium. Cells will be counted and plated at 3 x 10^6 cells per T25 flask for establishment of primary NB cells in culture, or use directly for ODC activity assay and associated protein detection by Western blotting as described below.

ODC Activity Measurement. The ODC enzyme activity in NB tumor tissue or bone marrow-derived primary NB cells will be determined using a standard assay as

previously reported[2]. In brief, primary NB cells will be washed twice with ice-cold PBS and harvested in 50 mM sodium phosphate buffer (pH 7.2), 0.1mM EDTA, 2.5mM DTT, supplemented with 1x protease inhibitor cocktail (Roche Molecular Biochemicals, Indianapolis, IN) on ice. Cells will be transferred immediately to Eppendorf tubes and snap-frozen in liquid nitrogen and lysed by three freeze/thaw cycles and cell lysates prepared by centrifugation for 20 min at 14,000 rpm and 4°C. Homogenized tumor tissues will be prepared as described above using the same lysis buffer. ODC activity will be measured in a reaction mixture that contains 20 μM L-[1-^{14}C]ornithine (47.70 mCi/mmol; NEN Life Science Products, Boston, MA) and cell extract of NB cells. The release of $^{14}CO2$ in response to ODC enzyme activities in cell extracts will be trapped in potassium hydroxide and measured in a liquid scintillation counter. Total protein amounts of cell extracts will be determined by Bradford assay[3] and used to determine specific enzyme activities.

Urinary dcSAM:

Adenine and its derivatives are known to react with 2-chloroacetaldehyde to form highly fluorescent tricyclic derivatives. This reaction gives a sensitive and specific method for measuring dc-SAM in urine and plasma samples. The reaction mixture will be incubated at 40°C overnight. An aliquot of this mixture will be injected onto an Altex Ultrosphere column for chromatographic separation. Detection will be accomplished using a Perkin-Elmer LS-4 spectrofluorometer, as described by others.[77]

Urinary Polyamines and Prostaglandins

High performance liquid chromatography (HPLC) methods will be used, as per previous studies ([4, 77]), to detect any molecule with a free primary amine including putrescine, spermidine, spermine, monoacetylspermidine and monoacetylspermine. Samples will be adjusted to 0.2N perchloric acid and analyzed directly. Acid hydrolysis methods will be employed to remove acetyl groups, and thus measure diacetylated amines. The detection level will be 1-10 pmol. Sources of error associated with these measures in colonic tissue have been previously reported ([78]). Urinary creatinine levels will also be determined, using a commercial kit (Oxford Biochemical Research), to normalize urinary polyamines. In the method, picric acid reacts with creatinine and other urinary components to produce an orange color, which can be quantified spectrophotometrically at 490 nm at alkaline pH. The creatinine reaction degrades rapidly when acidified. The difference in optical density is a direct measure of the creatinine concentration.

14.1.1 Optional Bone Marrow Collection

If subject agrees to optional biology portion of study, additional bone marrow samples should be sent at the following time points:

All Subjects- Enrollment

For Bone Marrow Positive subjects only- also send samples at end of cycle 1, 3, 5, and every other cycle after, and at End of Protocol Therapy

Sample Collection-

Send 1 green top (heparin) tube to each of the following sites with a minimum of 2cc and preferably 5cc of bone marrow aspirate in them. Can be shipped room temperature- but needs to be sent out same day priority overnight (must get there within 24 hours of the draw). Shipments are only accepted Monday through Friday, so Bone Marrow draw needs to be done and shipped out on Monday through Thursday only please.

8.5.2 Bone marrow 2-5cc in heparin tube at room temperature and will be shipped FED EX overnight to:

One tube will go to
Ping Zhao
Neuroblastoma Translational Research Laboratory
Van Andel Research Institute -5th floor
333 Bostwick Ave
Grand Rapids, MI 49503
Ph: 616-234-5394
ping.zhao@vai.org

The other tube will go to:
Flow Cytometry Lab of Spectrum Health
Attn: John Roys
Lemmen-Holton Cancer Pavilion
145 Michigan Street NE Suite 6201
Grand Rapids, MI 49503
phone: 616-486-6286
John.roys@spectrum-health.org

Appendix I: Performance Status/Scores

Performance Status Criteria
Karnofsky and Lansky performance scores are intended to be multiples of 10

ECOG (Zubrod)		Karnofsky		Lansky*	
Score	Description	Score	Description	Score	Description
0	Fully active, able to carry on all pre-disease performance without restriction.	100	Normal, no complaints, no evidence of disease	100	Fully active, normal.
		90	Able to carry on normal activity, minor signs or symptoms of disease.	90	Minor restrictions in physically strenuous activity.
1	Restricted in physically strenuous activity but ambulatory and able to carry out work of a light or sedentary nature, e.g., light housework, office work.	80	Normal activity with effort; some signs or symptoms of disease.	80	Active, but tires more quickly
		70	Cares for self, unable to carry on normal activity or do active work.	70	Both greater restriction of and less time spent in play activity.
2	Ambulatory and capable of all self-care but unable to carry out any work activities. Up and about more than 50% of waking hours	60	Required occasional assistance, but is able to care for most of his/her needs.	60	Up and around, but minimal active play; keeps busy with quieter activities.
		50	Requires considerable assistance and frequent medical care.	50	Gets dressed, but lies around much of the day; no active play, able to participate in all quiet play and activities.
3	Capable of only limited self-care, confined to bed or chair more than 50% of waking hours.	40	Disabled, requires special care and assistance.	40	Mostly in bed; participates in quiet activities.
		30	Severely disabled, hospitalization indicated. Death not imminent.	30	In bed; needs assistance even for quiet play.
4	Completely disabled. Cannot carry on any self-care. Totally confined to bed or chair.	20	Very sick, hospitalization indicated. Death not imminent.	20	Often sleeping; play entirely limited to very passive activities.
		10	Moribund, fatal processes progressing rapidly.	10	No play; does not get out of bed.

*The conversion of the Lansky to ECOG scales is intended for NCI reporting purposes only.

References

1. Ater JL, Gardner KL, Foxhall LE, Therrell BL, Jr., Bleyer WA. Neuroblastoma screening in the United States: results of the Texas Outreach Program for neuroblastoma screening. Cancer 1998; 82(8):1593-602.

2. Bosslet K, Mennel HD, Rodden F, et al. Monoclonal antibodies against epitopes on ganglioside GD2 and its lactones. Markers for gliomas and neuroblastomas. Cancer Immunol Immunother 1989; 29(3):171-8.

3. Cheung NK, Kushner BH, Yeh SD, Larson SM. 3F8 monoclonal antibody treatment of patients with stage 4 neuroblastoma: a phase II study. Int J Oncol 1998; 12(6):1299-306.

4. Harras A. Cancer Rates and Risks. National Cancer Institute, NIH Publication. 1996:96-691.

5. Brodeur GM. Neuroblastoma: biological insights into a clinical enigma. Nat Rev Cancer 2003; 3(3):203-16.

6. Seeger RC, Brodeur GM, Sather H, et al. Association of multiple copies of the N-myc oncogene with rapid progression of neuroblastomas. N Engl J Med 1985; 313(18):1111-6.

7. Ben-Yosef T, Yanuka O, Halle D, Benvenisty N. Involvement of Myc targets in c-myc and N-myc induced human tumors. Oncogene 1998; 17(2):165-71.

8. Slack A, Chen Z, Tonelli R, et al. The p53 regulatory gene MDM2 is a direct transcriptional target of MYCN in neuroblastoma. Proc Natl Acad Sci U S A 2005 Jan 18; 102(3):731-6. Epub 2005 Jan 11.

9. Marton LJ, Pegg AE. Polyamines as targets for therapeutic intervention. Annu Rev Pharmacol Toxicol 1995; 35:55-91.

10. Auvinen M, Paasinen A, Andersson LC, Holtta E. Ornithine decarboxylase activity is critical for cell transformation. Nature 1992; 360(6402):355-8.

11. Ernestus RI, Rohn G, Schroder R, et al. Polyamine metabolism in brain tumours: diagnostic relevance of quantitative biochemistry. J Neurol Neurosurg Psychiatry 2001; 71(1):88-92.

12. Mohan RR, Challa A, Gupta S, et al. Overexpression of ornithine decarboxylase in prostate cancer and prostatic fluid in humans. Clin Cancer Res 1999;5(1):143-7.

13. Lu X, Pearson A, Lunec J. The MYCN oncoprotein as a drug development target. Cancer Lett 2003; 197(1-2):125-30.

14. Lutz W, Stohr M, Schurmann J, Wenzel A, Lohr A, Schwab M. Conditional expression of N-myc in human neuroblastoma cells increases expression of alpha-prothymosin and ornithine decarboxylase and accelerates progression into S-phase early after mitogenic stimulation of quiescent cells. Oncogene 1996; 13(4):803-12.

15. Mamont PS, Duchesne MC, Grove J, Bey P. Anti-proliferative properties of DL-alpha-difluoromethyl ornithine in cultured cells. A consequence of the irreversible inhibition of ornithine decarboxylase. Biochem Biophys Res Commun 1978; 81(1):58-66.

16. Porter CW, Regenass U, Bergeron RJ. Polyamine inhibitors and analogs as potential anticancer agents. In: Dowling RH, Folsch UR, Loser C, editors. Falk symposium on polyamines in the gastrointestinal tract. Dordrecht, the Netherlands: Kluwer Academic Publishers; 1992. p. 301-22.

17. Seiler N, Atanassov CL, Raul F. Polyamine metabolism as target for cancer chemoprevention (review). Int J Oncol 1998; 13(5):993-1006.

18. McCann PP, Pegg AE. Ornithine decarboxylase as an enzyme target for therapy. Pharmacol Ther 1992; 54(2):195-215.

19. Marton LJ, Morris DR. Molecular and cellular functions of polyamines. In: McCann PP, Pegg AE, Sjoerdsma A, editors. Inhibition of Polyamine Metabolism: Biological Significance and Basis for New Therapies. New York: Academic Press; 1987. p. 79-105.

20. Fuller DJ, Gerner EW, Russell DH. Polyamine biosynthesis and accumulation during the G1 to S phase transition. J Cell Physiol 1977; 93(1):81-8.

21. Kaczmarek L, Calabretta B, Ferrari S, de Riel JK. Cell-cycle-dependent expression of human ornithine decarboxylase. J Cell Physiol 1987; 132(3):545-51.

22. Kahana C, Nathans D. Isolation of cloned cDNA encoding mammalian ornithine decarboxylase. Proc Natl Acad Sci U S A 1984; 81(12):3645-9.

23. Bello-Fernandez C, Packham G, Cleveland JL. The ornithine decarboxylase gene is a transcriptional target of c-Myc. Proc Natl Acad Sci U S A 1993; 90(16):7804-8.

24. Pena A, Reddy CD, Wu S, et al. Regulation of human ornithine decarboxylase expression by the c-Myc.Max protein complex. J Biol Chem 1993; 268(36):27277-85.

25. Wagner AJ, Meyers C, Laimins LA, Hay N. c-Myc induces the expression and activity of ornithine decarboxylase. Cell Growth Differ 1993; 4(11):879-83.

26. Heby O, Persson L. Molecular genetics of polyamine synthesis in eukaryotic cells. Trends Biochem Sci 1990; 15(4):153-8.

27. Metcalf BW, Bey P, Danzin C, Jung MJ, Casara P, Vevert JP. Catalytic irreversible inhibition of mammalian ornithine decarboxylase (E.C. 4.1.1.17) by substrate and product analogs. J Am Chem Soc 1978; 100:2551-3.

28. Poulin R, Lu L, Ackermann B, Bey P, Pegg AE. Mechanism of the irreversible inactivation of mouse ornithine decarboxylase by alpha-difluoromethylornithine. Characterization of sequences at the inhibitor and coenzyme binding sites. J Biol Chem 1992; 267(1):150-8.

29. Meyskens FL, Jr., Gerner EW. Development of difluoromethylornithine (DFMO) as a chemoprevention agent. Clin Cancer Res 1999; 5(5):945-51.

30. Chapman SK. Antitumor effects of vitamin A and inhibitors of ornithine decarboxylase in cultured neuroblastoma and glioma cells. Life Sci 1980; 26(16):1359-66.

31. Melino G, Farrace MG, Ceru MP, Piacentini M. Correlation between transglutaminase activity and polyamine levels in human neuroblastoma cells. Effect of retinoic acid and alpha-difluoromethylornithine. Exp Cell Res 1988; 179(2):429-45.

32. Pegg AE. Polyamine metabolism and its importance in neoplastic growth and a target for chemotherapy. Cancer Res 1988; 48(4):759-74.

33. Takahashi Y, Mai M, Nishioka K. alpha-difluoromethylornithine induces apoptosis as well as anti-angiogenesis in the inhibition of tumor growth and metastasis in a human gastric cancer model. Int J Cancer 2000; 85(2):243-7.

34. Carbone PP, Pirsch JD, Thomas JP, et al. Phase I chemoprevention study of difluoromethylornithine in subjects with organ transplants. Cancer Epidemiol Biomarkers Prev 2001;10(6):657-61.

35. Eskens FA, Greim GA, van Zuylen C, et al. Phase I and pharmacological study of weekly administration of the polyamine synthesis inhibitor SAM 486A (CGP 48 664) in patients with solid tumors. European Organization for Research and Treatment of Cancer Early Clinical Studies Group. Clin Cancer Res 2000; 6(5):1736-43.

36. Fabian CJ, Kimler BF, Brady DA, et al. A phase II breast cancer chemoprevention trial of oral alpha-difluoromethylornithine: breast tissue, imaging, and serum and urine biomarkers. Clin Cancer Res 2002; 8(10):3105-17.

37. Levin VA, Hess KR, Choucair A, et al. Phase III randomized study of postradiotherapy chemotherapy with combination alpha-difluoromethylornithine-PCV versus PCV for anaplastic gliomas. Clin Cancer Res 2003; 9(3):981-90.

38. Levin VA, Uhm JH, Jaeckle KA, et al. Phase III randomized study of postradiotherapy chemotherapy with alpha-difluoromethylornithine-procarbazine, N-(2-chloroethyl)-N'-cyclohexyl-N-nitrosurea, vincristine (DFMO-PCV) versus PCV for glioblastoma multiforme. Clin Cancer Res 2000; 6(10):3878-84.

39. Paridaens R, Uges DR, Barbet N, et al. A phase I study of a new polyamine biosynthesis inhibitor, SAM486A, in cancer patients with solid tumours. Br J Cancer 2000 Sep; 83(5):594-601.

40. Pless M, Belhadj K, Menssen HD, et al. Clinical efficacy, tolerability, and safety of SAM486A, a novel polyamine biosynthesis inhibitor, in patients with relapsed or refractory non-Hodgkin's lymphoma: results from a phase II multicenter study. Clin Cancer Res 2004 Feb 15; 10(4):1299-305.

41. Siu LL, Rowinsky EK, Hammond LA, et al. A phase I and pharmacokinetic study of SAM486A, a novel polyamine biosynthesis inhibitor, administered on a daily-times-five every-three-week schedule in patients with Advanced solid malignancies. Clin Cancer Res 2002; 8(7):2157-66.

42. van Zuylen L, Bridgewater J, Sparreboom A, et al. Phase I and pharmacokinetic study of the polyamine synthesis inhibitor SAM486A in combination with 5-fluorouracil/leucovorin in metastatic colorectal cancer. Clin Cancer Res 2004 Mar 15; 10(6):1949-55.

43. Chopra S, Wallace HM. Induction of spermidine/spermine N1-acetyltransferase in human cancer cells in response to increased production of reactive oxygen species. Biochem Pharmacol 1998 Apr 1; 55(7):1119-23.

44. Wallace HM, Fraser AV, Hughes A. A perspective of polyamine metabolism. Biochem J 2003; 376(Pt 1):1-14.

45. Wang Y, Devereux W, Woster PM, Stewart TM, Hacker A, Casero RA, Jr. Cloning and characterization of a human polyamine oxidase that is inducible by polyamine analogue exposure. Cancer Res 2001 Jul 15; 61(14):5370-3.

46. Heby O. Role of polyamines in the control of cell proliferation and differentiation. Differentiation 1981; 19(1):1-20.

47. Pegg AE. Recent advances in the biochemistry of polyamines in eukaryotes. Biochem J 1986; 234(2):249-62.

48. Tabor CW, Tabor H. Polyamines. Annu Rev Biochem 1984; 53:749-90.

49. Chen KY, Nau D, Liu AY. Effects of inhibitors of ornithine decarboxylase on the differentiation of mouse neuroblastoma cells. Cancer Res 1983; 43(6):2812-8.

50. Melino G, Piacentini M, Patel K, Annicchiarico-Petruzzelli M, Piredda L, Kemshead JT. Retinoic acid and alpha-difluoromethylornithine induce different expression of neural-specific cell adhesion molecules in differentiating neuroblastoma cells. Prog Clin Biol Res 1991; 366:283-91.

51. Wainwright LJ, Lasorella A, Iavarone A. Distinct mechanisms of cell cycle arrest control the decision between differentiation and senescence in human neuroblastoma cells. Proc Natl Acad Sci U S A 2001; 98(16):9396-400.

52. Wallick CJ, Gamper I, Thorne M, et al. Key role for p27Kip1, retinoblastoma protein Rb, and MYCN in polyamine inhibitor-induced G1 cell cycle arrest in MYCN-amplified human neuroblastoma cells. Oncogene 2005 Aug 25;24(36):5606-18.

53. Koomoa DL, Yco LP, Borsics T, Wallick CJ, Bachmann AS. Ornithine decarboxylase inhibition by alpha-difluoromethylornithine activates opposing signaling pathways via phosphorylation of both Akt/protein kinase B and p27Kip1 in neuroblastoma. Cancer Res 2008 Dec 1; 68 (23):9825-31.

54. Hogarty MD, Norris MD, Davis K, et al. ODC1 is a critical determinant of MYCN oncogenesis and a therapeutic target in neuroblastoma. Cancer Res 2008 Dec 1; 68(23):9735-45.

55. Rounbehler RJ, Li W, Hall MA, Yang C, Fallahi M, Cleveland JL. Targeting ornithine decarboxylase impairs development of MYCN-amplified neuroblastoma. Cancer Res 2009; 69(2):547-53.

56. AACR 2009, Abstract #3203

57. AACR 2009, Abstract #3208

58. Dorr RT, Liddil JD, Gerner EW. Modulation of etoposide cytotoxicity and DNA strand scission in L1210 and 8226 cells by polyamines. Cancer Res 1986 Aug; 46(8):3891-5.

59. Meyskens FL, Jr., McLaren CE, Pelot D, et al. Difluoromethylornithine plus sulindac for the prevention of sporadic colorectal adenomas: A randomized placebo-controlled, double-blind trial. Cancer Prev Res 2008; 1(1):32-8.

60. Boyle JO, Meyskens FL, Jr, Garewal HS, Gerner EW: Polyamine contents in rectal and buccal mucosae in humans treated with oral difluoromethylornithine. Cancer Epidemiology, Biomarkers & Prevention. 1:131-135, 1992

61. Meyskens, FL, Emerson SS, Pelot D, Meshkinpour L, et al.: Dose de-escalation chemoprevention trial of 2 difluoromethylornithine in patients with colon polyps. J Natl Cancer Inst 86: 1122-1130, 1994

62. Meyskens FL, Gerner E, Emerson S, Pelot D, Durbin T, Doyle K, Lagerberg W: A Randomized Double-Blind Placebo Controlled Phase IIb Trial of Difluoromethylornithine for Colon Cancer Prevention. Journal of the National Cancer Institute, 90(16)1212-1218,1998

63. Love RR, Jacoby R, Newton MA, Tutsch KD, Simon K, Pomplun M, Verma AK. A randomized, placebo-controlled trial of low-dose alpha-difluoromethylornithine in individuals at risk for colorectal cancer. Cancer Epidemiol Biomarkers Prev. 1998 Nov;7(11):989-92.

64. Croghan MK, Aickin MG, Meyskens FL. Dose-related alpha-difluoromethylornithine ototoxicity. Am J Clin Oncol. 1991 Aug;14(4):331-5.

65. Doyle KJ, McLaren CE, Shanks JE, Galus C, Meyskens FL Jr:. Effects of DFMO chemoprevention on audiometry thresholds and otoacoustic emissions. Arch. Otolaryngology Head and Neck Surgery, 127:553-558, 2001

66. Brant LJ, Gordon-Salant S, Pearson JD, Klein LL, Morrell CH, Metter EJ, Fozard JL. Risk factors related to age-associated hearing loss in the speech frequencies. J Am Acad Audiol. 1996 Jun;7(3):152-60.

67. Cruickshanks KJ, Tweed TS, Wiley TL, Klein BE, Klein R, Chappell R, Nondahl DM, Dalton DS. The 5-year incidence and progression of hearing loss: the epidemiology of hearing loss study. Arch Otolaryngol Head Neck Surg. 2003 Oct;129(10):1041-6.

68. Bradford MM. A rapid and sensitive method for the quantitation of microgram quantities of protein utilizing the principle of protein-dye binding. Anal Biochem. 1976 May 7;72:248-54.

69. Gilbert RS, Gonzalez GG, Hawel L 3rd, Byus CV. An ion-exchange chromatography procedure for the isolation and concentration of basic amino acids and polyamines from complex biological samples prior to high-performance liquid chromatography. Anal Biochem. 1991 Nov 15;199(1):86-92.

70. Wallick CJ, Gamper I, Thorne M, Feith DJ, Takasaki KY, Wilson SM, Seki JA, Pegg AE, Byus CV, Bachmann AS. Key role for p27Kip1, retinoblastoma protein Rb, and MYCN in polyamine inhibitor-induced G1 cell cycle arrest in MYCN-amplified human neuroblastoma cells. Oncogene. 2005 Aug 25;24(36):5606-18.

71. Inoue H, Fukunaga K, Munemura S, Tsuruta Y. Simultaneous determination of free and N-acetylated polyamines in urine by semimicro high-performance liquid chromatography using 4-(5,6-dimethoxy-2-phthalimidinyl)-2-methoxyphenylsulfonyl chloride as a fluorescent labeling reagent. Anal Biochem. 2005 Apr 15;339(2):191-7.

72. Schiavetti A, Varrasso G, Maurizi P, Cappelli C, Clerico A, Properzi E, Castello MA. Ten-day schedule oral etoposide therapy in advanced childhood malignancies J Pediatr Hematol Oncol. 22(2):119-24, 2000.

73. Kushner BH, Kramer K, Cheung NV, Oral Etoposide for Refractory and Relapsed Neuroblastoma, JCO, 17(10): 3221-3225, 1999.

74. Koomoa DT, Borsics T, Feith DJ, Coleman CS, Wallick CJ, Gamper I, Pegg AE, Bachmann AS: Inhibition of *S*-adenosylmethionine decarboxylase by the competitive inhibitor SAM486A connects polyamine metabolism with p53-Mdm2-Akt/PKB regulation and apoptotic cell death in neuroblastoma, Molecular Cancer Therapeutics, 8(7): 2067-2075, 2009.

75. Hogarty MD, Norris MD, Davis K, Liu X, Evageliou NF, Hayes CS, Pawel B, Guo R, Zhao H, Sekyere E, Keating J, Thomas W, Cheng NC, Murray J, Smith J, Sutton R, Venn N, London WB, Buxton A, Gilmour SK, Marshall GM, Haber M. ODC1 is a critical determinant of MYCN oncogenesis and a therapeutic target in neuroblastoma. Cancer Res. 2008 Dec 1;68(23):9735-45.

76. Therasse P, Arbuck SG, Eisenhauer EA, Wanders J, Kaplan RS, Rubinstein L, Verweij J, Van Glabbeke M, van Oosterom AT, Christian MC, Gwyther SG. New guidelines to evaluate the response to treatment in solid tumors. European Organization for Research and Treatment of Cancer, National Cancer Institute of the United States, National Cancer Institute of Canada. J Natl Cancer Inst. 2000 Feb 2;92(3):205-16.

77. Haegele, K.D., et al., Decarboxylated-S-adenosylmethionine excretion: a biochemical marker of ornithine decarboxylase inhibition by alpha-difluoromethylornithine. Cancer Res, 1987. 47(3): p. 890-5.

78. Hixson, L.J., et al., Sources of variability in estimating ornithine decarboxylase activity and polyamine contents in human colorectal mucosa. Cancer Epidemiol Biomarkers Prev, 1994. 3(4): p. 317-23.

79. Pasic TR, Heisey D, Love RR, alpha-difluoromethylornithine ototoxicity. Chemoprevention clinical trial results. Arch Otolaryngol Head Neck Surg. 1997 Dec;123(12):1281-6.

80. Geerts D, Koster J, Albert D, Koomoa DL, Feith DJ, Pegg AE, Volckmann R, Caron H, Versteeg R, Bachmann AS. The polyamine metabolism genes ornithine decarboxylase and antizyme 2 predict aggressive behavior in neuroblastomas with and without MYCN amplification. Int J Cancer. 2009 Dec 3. [Epub ahead of print]. PMID: 19960435 [PubMed - as supplied by publisher].
